# Evaluation of Adverse Events and the Impact on Health-Related Outcomes in Patients Undergoing Surgery for Metastatic Spine Tumors: Analysis of the Metastatic Tumor Research and Outcomes Network (MTRON) Registry Dataset

**DOI:** 10.1177/21925682251347247

**Published:** 2025-06-06

**Authors:** Giovanni Barbanti Brodano, Cristiana Griffoni, Francesca Salamanna, Luigi Emanuele Noli, Annalisa Monetta, Alessandro Luzzati, Alexander C. Disch, Aron Lazary, Ori Barzilai, Ilya Laufer, Ziya L. Gokaslan, Michael G. Fehlings, Jorrit-Jan Verlaan, Dean Chou, Laurence D. Rhines, John H. Shin, William G. J. Teixeira, Daniel M. Sciubba, Chetan Bettegowda, Raphaële Charest-Morin, Stefano Boriani, Tony Goldschlager, Michael H. Weber, Michelle J. Clarke, John E. O’Toole, Cordula Netzer, C. Rory Goodwin, Addisu Mesfin, Praveen V. Mummaneni, Nicolas Dea, Jeremy J. Reynolds, Arjun Sahgal, Charles G. Fisher, Alessandro Gasbarrini, AO Spine Knowledge Forum Tumor

**Affiliations:** 1 Department of Spine Surgery, 18509 IRCCS Istituto Ortopedico Rizzoli, Bologna, Italy; 2 Surgical Sciences and Technologies, 18509 IRCCS Istituto Ortopedico Rizzoli, Bologna, Italy; 3 419170 ISNB Istituto Delle Scienze Neurologiche di Bologna, Bologna, Italy; 4 46767 IRCCS Istituto Ortopedico Galeazzi, Milan, Italy; 5 39063 University Hospital Carl Gustav Carus at the TU Dresden, Dresden, Germany; 6 National Center for Spinal Disorders, Budapest, Hungary; 7 5803 Memorial Sloan-Kettering Cancer Center, New York, NY, USA; 8 12297 New York University Langone Health, New York, NY, USA; 9 Brown University, Warren Alpert School of Medicine, Providence, RI, USA; 10 Division of Neurosurgery and Spine Program, 7938 University of Toronto, Toronto, ON, Canada; 11 8124 University Medical Center Utrecht, Utrecht, The Netherlands; 12 21611 Columbia University Irving Medical Center, New York, NY, USA; 13 461926 The University of Texas MD Anderson Cancer Center, Houston, TX, USA; 14 2348 Massachusetts General Hospital, Boston, MA, USA; 15 215027 Instituto do Câncer do Estado de São Paulo, São Paulo, Brazil; 16 5799 Northwell Health, Manhasset, NY, USA; 17 1500 Johns Hopkins University School of Medicine, Baltimore, MD, USA; 18 8166 University of British Columbia, Vancouver, BC, Canada; 19 9296 University of Bologna, Bologna, Italy; 20 2541 Monash University, Melbourne, VIC, Australia; 21 Department of Orthopedic Surgery, 7712 University of Connecticut, Farmington, CT, USA; 22 Department of Neurologic Surgery, 6915 Mayo Clinic, Rochester, MN, USA; 23 2468 Rush University Medical Center, Chicago, IL, USA; 24 30262 Universitätsspital Basel, Basel, Switzerland; 25 Spine Division, Department of Neurosurgery, Duke Center for Brain and Spine Metastasis, 609772 Duke University Medical Center, Durham, NC, USA; 26 Medstar Orthopaedic Institute, 12230 Georgetown University School of Medicine, Washington, DC, USA; 27 8785 University of California, San Francisco, CA, USA; 28 6397 Oxford University Hospitals NHS Trust, Oxford, UK; 29 71545 Sunnybrook Health Sciences Center, Toronto, ON, Canada; 30 Department of Biomedical and Neuromotor Sciences, 9296 Alma Mater Studiorum University of Bologna, Bologna, Italy

**Keywords:** spinal metastases, surgical treatment, adverse events, complications, survival, length of stay, quality of life

## Abstract

**Study Design:**

This study is part of the AO Spine Metastatic Tumor Research and Outcomes Network [MTRON], an international multicenter prospective observational registry including patients with spinal metastases.

**Objectives:**

This study aims to elucidate the incidence of surgical complications, their risk factors and consequent effects on survival outcomes, hospital length of stay, and overall health-related quality of life (HRQOL) parameters in a large cohort of patients affected by spinal metastases who were surgically treated.

**Methods:**

Available data from February 2017 to July 2023 were analyzed. The primary outcome of this study was the evaluation of the incidence of intraoperative and postoperative adverse events (AEs). The secondary outcomes included the assessment of risk factors for surgery-related AEs and the impact of AEs on survival, length of hospital stay and quality of life.

**Results:**

Among the 1267 patients analyzed, 6.9% experienced intraoperative AEs and 19.3% experienced at least 1 postoperative AE. Several factors resulted to be associated to the occurrence of postoperative AEs: age, smoking habit, poor Eastern Cooperative Oncology Group (ECOG) Performance status, previous radiation therapy at the index target, duration of surgery, number of instrumented levels, simultaneous anterior and posterior approach, presence of metastases at other sites, multiple spinal metastases. Postoperative AEs were associated with reduced survival rates, increased hospital length of stay and poorer HRQOL outcomes, particularly in domains such as neurological function and mental health. In general, surgery substantially improves HRQOL across multiple domains, with these benefits persisting over time despite the occurrence of AEs. However, patients with preoperative risk factors, including comorbidities, smoking, neurological impairment, and prior radiation therapy, experienced less improvement.

**Conclusions:**

The negative impact of AEs on overall survival and HRQOL could be associated with the presence of some preoperative parameters of frailty that are detected as risk factors for AEs occurrence. This finding emphasizes the need for personalized preoperative assessments and optimized perioperative care strategies.

## Introduction

Recent years have witnessed a notable rise in the incidence of spinal metastases, attributed to advancements in cancer therapeutics and prolonged patient survival.^1^ Collectively, spinal metastases afflict 70% of cancer patients, with 10% to 20% experiencing skeletal-related events, including refractory pain, pathological vertebral fractures, and spinal cord compression, culminating in neurological impairment, compromised ambulatory function, diminished quality of life, and reduced survival rates.^2-4^ Accordingly, surgical intervention is often warranted to alleviate symptoms, restore functional integrity and improve health-related quality of life (HRQOL).^5,6^

Surgical treatments for spinal metastases encompass palliative resections employing anterior, posterior, or anteroposterior approaches in general, and are often accompanied by conventional or stereotactic radiation.^7^ Less invasive techniques, such as percutaneous stabilization and cement augmentation, are also utilized regularly. Given the primarily palliative nature of treatment and the considerable vulnerability of these patients, surgical interventions carry a significant risk of complications and thus decision making is complex.^7^ Various studies have reported complication rates ranging from 10% to 76.2% following spine surgery for metastases.^8-27^ In a retrospective study of 987 patients, the overall complication rate during the index hospital admission/surgery and subsequent admissions was 39%, with 27% experiencing one major complication and 12% encountering 2 or more postoperative complications.^22^ A large administrative database retrospective study, inherently providing less detail, involving 26,233 patients reported an in-hospital complication rate of 21.9%.^15^ A more recent systematic literature review indicated a 29% risk of complications associated with spine surgery for metastases, with infections and pulmonary complications being the most prevalent.^23^ Finally, a rigorous prospective study on complications in emergency surgery for metastatic spine diseases reported a very high rate of complications, with 76.2% of patients experiencing at least 1 adverse event (AE).^27^ Complications lead to prolonged hospital stays, additional diagnostic tests, treatments, and the need for rehabilitation services. Importantly, AEs in this patient population may also interrupt or delay systemic or radiation therapy. Furthermore, readmission rates within 3 months post-discharge range from 9.7% to 16.8%, with corresponding rates of reoperations for complications.^12,22^ This AE profile contributes to significant healthcare costs and suffering to patients with oftentimes limited life expectancy.

Making inroads to reduce complications depends on first improving data quality and then acting on these data. The extreme variability in complication rates displayed in the literature and thus inaccuracy rests almost entirely on the process of the surveillance, capture, reporting and research methodology; an administrative vs research registry, dedicated surveillance personal, standardized psychometrically sound classification and severity grading, retrospective vs prospective collection and detailed follow-up. Furthermore, multicenter and multicultural collection is essential to optimize generalizability.

The Metastatic Tumor Research and Outcomes Network (MTRON) (National Clinical Trial n. NCT02830451) is part of the AO Spine Knowledge Forum Tumor’s global network of surgeons and oncologists dedicated to improving care and outcomes in these unique and deserving patients. MTRON has acted on many of the afore mentioned limitations inherent to the literature by developing a comprehensive prospective, multicenter, multicountry registry of patients who have undergone surgical intervention for spine metastases. Utilizing this registry the primary outcome of this study is to determine the incidence of AEs in patients with spine metastases requiring surgery with or without radiation. Secondary outcomes are first to assess the relationship of complications to survival, hospital length of stay, and overall HRQOL and second to identify risk factors for developing complications in this patient cohort.

## Methods

### Study Design

This study is part of the AO Spine Knowledge Forum Tumor’s Metastatic Tumor Research and Outcomes Network [MTRON], an international multicenter prospective observational registry including patients with spinal metastases. The trial is registered at ClinicalTrials.gov with ID number NCT02830451.

Each participating center’s local Ethics board approved the study protocol and IRB approval numbers for each center are reported in the title page.

Patients were eligible to be enrolled into the registry if they were 18 years or older and were diagnosed with a metastatic spinal tumor. All patients signed a study-specific informed consent before the enrolment for the study.

For this predefined research question, patients were eligible for inclusion in the analysis if they were prospectively treated with surgery for metastatic spine disease. Available data from February 2017 to July 2023 were analyzed.

### Outcome Measures

Prospectively collected data included the following: demographic and clinical data at baseline, number and location of spinal lesions, primary tumor location, histology and subtype, presence of other distant metastases, prior treatments of index metastasis.

Baseline functional status was assessed with preoperative American Spinal Injury Association (ASIA) for neurological assessment and Eastern Cooperative Oncology Group (ECOG) for performance status. The degree of spinal cord compression and instability were assessed by Bilsky grade epidural compression classification and Spinal Instability Neoplastic Score (SINS) at the time of treatment, respectively. Surgical and radiotherapy treatment details were collected. Pain was assessed at baseline and at all follow-up visits with the pain NRS. Health-related Quality of Life (HRQOL) metrics included the Spine Oncology Study Group Outcomes Questionnaire (SOSGOQ2.0)^28^ and Euro-QoL 5D (EQ-5D-3L),^29^ which were performed at baseline and all follow-up visits. Surgery-related AEs were collected using the Spine Adverse Events Severity system version 2 (SAVES V2) and were classified as minor if grade was ≤3 and severe if grade was >3.^30^

The primary outcome of this study was the evaluation of the incidence of intraoperative and postoperative AEs, in patients requiring surgical intervention for spinal metastases. The secondary outcomes included the impact of surgery-related AEs on survival, length of hospital stay and HRQOL. All patients’ data were collected and stored in a de-identified format in a secure web-based database [REDCap v6.5.2, Vanderbilt University, Nashville, TN, USA]. Only patients who had at least half of the digital forms completed in the REDCap database were included in the analysis to ensure data quality.

### Statistical Analysis

Descriptive statistics were used to summarize baseline population characteristics, including demographic variables, stratified by the occurrence and severity of AEs. For the primary endpoint—incidence of intraoperative and postoperative AEs—percentages with 95% confidence intervals (CIs) were reported. Subjects experiencing multiple AEs of the same type were counted once.

For secondary endpoints, such as survival and length of stay, Kaplan-Meier analysis was employed to compare patient groups with and without AEs. Differences in survival between these groups were assessed using a stratified log-rank test. To adjust for covariates, we applied the Cox proportional hazards model to estimate hazard ratios (HRs) with their corresponding 95% CIs.

Longitudinal data, such as repeated measurements of patient-reported outcomes across timepoints, were analyzed using a mixed model for repeated measures (MMRM). Model estimates were presented with their associated 95% CIs. Additionally, lasso regression was conducted to explore the relationships between potential covariates (eg, age, ECOG performance status, smoking history) and AE occurrence, identifying key predictors. The final model included selected variables along with their coefficients.

Missing data was addressed using multiple imputation techniques. A two-sided *P*-value of less than 0.05 was considered statistically significant. All statistical analyses were performed using SAS (version 9.4, SAS Institute Inc, Cary, NC, USA) and R (version 4.1.2 or later, R Core Team, 2021).

## Results

### Descriptive Analysis of the Study Population

The overall study cohort of 1267 patients contained 566 female (44.7%) and 701 male (55.3%) patients with a mean age of 62 years (range 18-89). Baseline patients’ data, tumor characteristics and previous treatments for the index target are reported in Table 1.

**Table 1.** Summary of Patients’ Data, Tumor Variables and Previous Treatments for the Index Target.

Baseline Characteristic	Total Number of Patients = 1267
Gender	N (%)
Female	566 (44.67)
Male	701 (55.33)
Age (years)	Mean (range)
	62.03 (18-89)
Charlson comorbidity index	Mean (range)
	6.44 (2-15)
Was smoking status assessed? n (%)	1266
No	315 (24.88)
Yes	951 (75.12)
Does the patient smoke or use chewing tobacco? n (%)	
No	593 (62.42)
Yes- currently	100 (10.53)
Yes- previously	257 (27.05)
Location of the index target	N (%)
Cervical	258 (20.4)
Thoracic	890 (70.2)
Lumbar	568 (44.8)
Sacral	139 (11.0)
Other spinal metastases	N (%)
Yes	723 (57.1)
No	544 (42.9)
Visceral metastases	N (%)
Yes	467 (36.9)
No	800 (63.1)
Previous surgery to treat the index target	N (%)
Yes	114 (9.00)
No	1153 (91.00)
Previous radiation therapy to treat the index target	N (%)
Yes	304 (24.01)
No	962 (75.99)
Previous systemic therapy to treat the index target	N (%)
Yes	320 (25.28)
No	946 (74.72)

**Figure 1.**
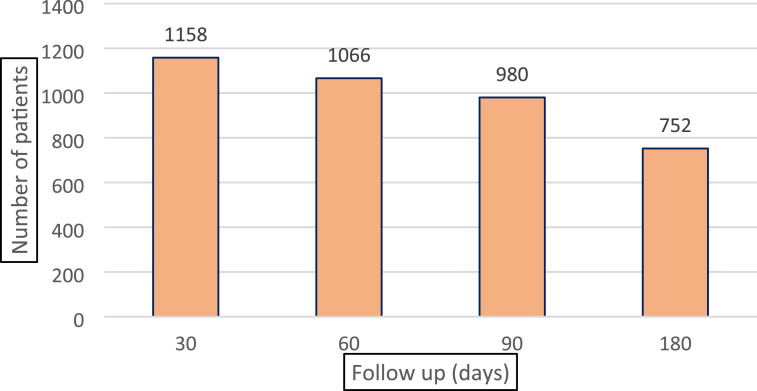
Number of patients at follow-up at 1 month, 2 months, 3 months, and 6 months after surgery.

As shown in Figure 1, 1158 patients (90.8%) were followed up to 30 days after surgery, 1066 patients (84.1%) were followed up to 60 days (2 months) after surgery and 752 patients (59.4%) to 180 days (6 months) after surgery.

**Figure 2.**
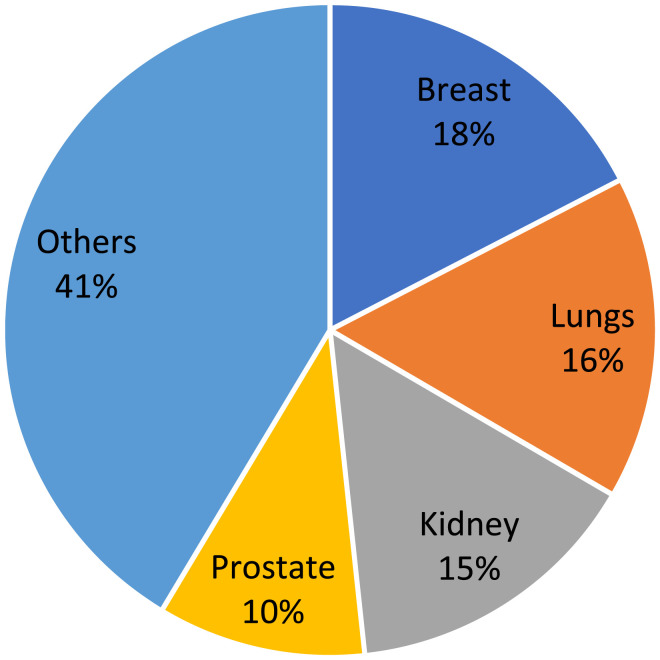
Type of primary tumors in the overall cohort of 1267 patients.

The distribution of primary tumors is shown in Figure 2. Most of metastatic index targets were in the thoracic spine (890, 70.2%); other spinal metastases were present in 723 patients (57.1%) and visceral metastases were present in 467 patients (36.9%). 114 patients (9%) were previously treated with surgery at the index target, while 304 patients (24.0%) underwent previous radiation therapy at the index target, and 320 patients (25.3%) underwent previous systemic therapy.

Baseline data concerning the neurological and performance status, the epidural spinal cord compression and the spinal instability are reported in Table 2. Neurological evaluation by ASIA score was assessed in 95.1% of patients; 90.3% of patients for which an ASIA score was determined had no neurological damage or mild neurological damage (ASIA score D/E), while Epidural Spinal Cord Compression (ESCC) was detected in most patients (1210, 95.5%) with a high grade (≥2) in 736 patients (60.8%). Concerning the ECOG performance status, 299 patients (23.8%) presented a poor score (ECOG 3-4); the SINS score showed potential instability (score 7- 12) in 810 patients (66.2%) and 279 patients (22.8%) presented with definite spinal instability (SINS ≥13).

**Table 2.** Summary of Neurological Variables, Epidural Spinal Cord Compression, Performance Status and Instability Evaluation.

Baseline Characteristic	Total Number of Patients = 1267
Has ASIA score been assessed? n (%)	1267
No	62 (4.89)
Yes	1205 (95.11)
ASIA impairment scale, n (%)	1200
A/B/C	117 (9.75)
D/E	1083 (90.25)
Have symptoms been assessed? n (%)	1267
No	3 (0.24)
Yes	1264 (99.76)
Epidural spinal cord compression for most severe compression of index target? n (%)	1210
Low: 0-1	474 (39.17)
High: 2-3	736 (60.83)
ECOG performance status, n (%)	1257
0	163 (12.97)
1/2	795 (63.25)
3/4	299 (23.79)
5	0 (0.00)
Has SINS score been assessed? n (%)	1267
No	25 (1.97)
Yes	1242 (98.03)
Total SINS score, n (%)	1224
Stable: 0-6	135 (11.03)
Indeterminate: 7-12	810 (66.18)
Unstable: 13-18	279 (22.79)

Surgical variables are reported in Table 3. Most patients were treated with a posterior approach (1199, 94.8%) and surgery was usually performed in a single stage (97.6% of cases). The mean surgical time was 3.4 ± 1.9 hours, and the mean number of instrumented levels was 4.8 ± 2.8.

**Table 3.** Summary of Surgical Variables.

Baseline Characteristic	Total Number of Patients = 1267
Surgical approach within this first surgical stage, n (%)	1265
Anterior	45 (3.56)
Posterior	1199 (94.78)
Both (simultaneous)	21 (1.66)
Number of stages, n (%)	1266
1	1236 (97.63)
2/3	30 (2.37)
Duration of first surgery (hours) n	1254
Mean (SD)	3.43 (1.86)
Median (Q1; Q3)	3.05 (2.20; 4.08)
Min; max	0.17; 13.13
Number of instrumented levels n	1267
Mean (SD)	4.80 (2.78)
Median (Q1; Q3)	5.00 (3.00; 6.00)
Min; max	0.00; 20.00
Surgical indication//Neurological: Functional radiculopathy, n (%)	1267
No	743 (58.64)
Yes	524 (41.36)
Surgical indication//Neurological: Myelopathy, n (%)	1267
No	949 (74.90)
Yes	318 (25.10)
Surgical indication//Neurological: High grade ESCC, n (%)	1267
No	764 (60.30)
Yes	503 (39.70)
Surgical indication//Oncologic: RT resistant, n (%)	1267
No	1087 (85.79)
Yes	180 (14.21)
Surgical indication//Oncologic: Best known treatment, n (%)	1267
No	452 (35.67)
Yes	815 (64.33)
Surgical indication//Stability, n (%)	1166
Stable	239 (20.50)
Impending instability	450 (38.59)
Unstable	477 (40.91)
Procedure type, n (%)	1245
Palliative procedure	858 (68.92)
Curettage	299 (24.02)
En bloc resection	88 (7.07)

Most of the surgical procedures (68.9%) had palliative intent, debulking was performed in 24.0% of cases and en-bloc resection in 7.1% of cases.

### Adverse Events Collection

The intraoperative AEs are reported in Table 4. Eighty-seven patients (6.9%) experienced at least 1 AE during the surgery and the most frequent AE was dural tears which occurred in 44 cases (3.5%). Most complications (80, 92%) had a severity grade ≤3 and were considered minor AEs.

**Table 4.** Intraoperative Adverse Events (Patient Level), Worst Severity Grade and Severity Group.

	N° tot = 1267		Severity Grade, n (%)						Severity Group, n (%)	
Adverse Events	N^a^	%^b^(95% CI^c^)	1	2	3	4	5	6	Minor	Major
Any intraoperative adverse event	87	6.9 (5.5; 8.4)	23 (26.4)	41 (47.1)	16 (18.4)	2 (2.3)	4 (4.6)	1 (1.1)	80 (92)	7 (8.0)
Allergic reaction	1	0.1 (0.0; 0.4)	1 (100.0)						1 (100.0)	0 (0.0)
Anesthesia related	1	0.1 (0.0; 0.4)	1 (100.0)						1 (100.0)	0 (0.0)
Bone implant interface failure requiring revision	1	0.1 (0.0; 0.4)			1 (100.0)				1 (100.0)	0 (0.0)
Cardiac	4	0.3 (0.1; 0.8)	1 (25.0)		2 (50.0)		1 (25.0)		3 (75.0)	1 (25.0)
Cord injury	2	0.2 (0.0; 0.6)	1 (50.0)				1 (50.0)		1 (50.0)	1 (50.0)
Dural tear	44	3.5 (2.5; 4.6)	14 (31.8)	25 (56.8)	4 (9.1)	1 (2.3)			43 (97.7)	1 (2.3)
Hardware malposition requiring revision	5	0.4 (0.1; 0.9)		1 (20.0)	4 (80.0)				5 (100.0)	0 (0.0)
Hypotension (systemic less than 85 mmHg for 15 min)	1	0.1 (0.0; 0.4)		1 (100.0)					1 (100.0)	0 (0.0)
Massive blood loss (Greater than 5 L in 24 h or greater than 2 L in 3 h)	12	0.9 (0.5; 1.6)		7 (58.3)	4 (33.3)		1 (8.3)		11 (91.7)	1 (8.3)
Nerve root injury	5	0.4 (0.1; 0.9)	2 (40.0)	1 (20.0)	1 (20.0)	1 (20.0)			4 (80.0)	1 (20.0)
Pressure sores	0	0.0 (0.0; 0.3)							0 (−)	0 (−)
Vascular injury	0	0.0 (0.0; 0.3)							0 (−)	0 (−)
Ventilation/airway	1	0.1 (0.0; 0.4)	1 (100.0)						1 (100.0)	0 (0.0)
Visceral injury	1	0.1 (0.0; 0.4)		1 (100.0)					1 (100.0)	0 (0.0)
Any other intraop AE	13	1.0 (0.5; 1.7)	4 (30.8)	7 (53.8)			1 (7.7)	1 (7.7)	11 (84.6)	2 (15.4)

^a^Number of patients with at least 1 AE. If a patient experienced multiple AEs under any AE class, the patient was only counted once.

^b^Estimated risk of developing at least 1 AE (calculated by dividing the number of patients experiencing at least 1 AE by the total number of patients).

^c^Confidence intervals for percentages were calculated using the exact method (Clopper Pearson method).

Note. All intraoperative AEs which happen after the first documented surgery to treat index lesion were taken into consideration. If a second surgery was performed the subsequent reported intraoperative AEs were not taken into consideration. If more than 1 event of the same type occurred for the same patient, the worst category has been used. The Adverse Event Severity system version 2 (SAVES V2) AE abstraction tool was used to record the morbidity data.

Postoperative AEs occurring during the hospital stay or during the follow-up period, are reported in Table 5. A total of 245 patients (19.3%) experienced at least 1 postoperative AE. Most postoperative AEs (95, 7.5%) were classified as “others” according to SAVES v2 system; deep wound infection (40 cases, 3.2%), wound dehiscence (32 cases, 2.5%) and urinary infection (31 cases, 2.4%) were the most frequent causes of postoperative AEs. Most postoperative complications (209, 85.7%) had a severity grade ≤3 (minor AEs).

**Table 5.** Postoperative Adverse Events (Patient Level), Worst Severity Grade and Severity Group.

	N° tot = 1267		Severity Grade, n (%)						Severity Group, n (%)	
Adverse Events	N^a^	%^b^(95% CI^c^)	1	2	3	4	5	6	Minor	Major
Any postoperative adverse event	245	19.3 (17.2; 21.)	28 (11.5)	98 (40.2)	83 (34.0)	19 (7.8)	5 (2.0)	11 (4.5)	209 (85.7)	35 (14.3)
Cardiac arrest/failure/arrhythmia	21	1.7 (1.0; 2.5)	3 (14.3)	7 (33.3)	5 (23.8)	2 (9.5)	1 (4.8)	3 (14.3)	15 (71.4)	6 (28.6)
Construct failure with loss of correction	4	0.3 (0.1; 0.8)		1 (25.0)	2 (50.0)	1 (25.0)			3 (75.0)	1 (25.0)
Construct failure without loss of correction	3	0.2 (0.0; 0.7)	1 (33.3)		2 (66.7)				3 (100.0)	0 (0.0)
CSF leak/meningocele	3	0.2 (0.0; 0.7)	1 (33.3)		1 (33.3)		1 (33.3)		2 (66.7)	1 (33.3)
Deep vein thrombosis	17	1.3 (0.8; 2.1)	1 (6.3)	9 (56.3)	5 (31.3)	1 (6.3)			15 (93.8)	1 (6.3)
Deep wound infection	40	3.2 (2.3; 4.3)	2 (5.0)	6 (15.0)	25 (62.5)	5 (12.5)	2 (5.0)		33 (82.5)	7 (17.5)
Delirium	15	1.2 (0.7; 1.9)	5 (33.3)	7 (46.7)	2 (13.3)	1 (6.7)			14 (93.3)	1 (6.7)
Dysphagia	11	0.9 (0.4; 1.5)	5 (45.5)	6 (54.5)					11 (100.0)	0 (0.0)
Dysphonia	2	0.2 (0.0; 0.6)	1 (50.0)	1 (50.0)					2 (100.0)	0 (0.0)
Gastrointestinal bleeding	5	0.4 (0.1; 0.9)	1 (20.0)		2 (40.0)			2 (40.0)	3 (60.0)	2 (40.0)
Hematoma	10	0.8 (0.4; 1.4)	3 (30.0)		5 (50.0)		2 (20.0)		8 (80.0)	2 (20.0)
Myocardial infarction	0	0.0 (0.0; 0.3)							0 (−)	0 (−)
Neurologic deterioration (greater equal 1 motor grade in ASIA motor scale)	10	0.8 (0.4; 1.4)		6 (60.0)	3 (30.0)	1 (10.0)			9 (90.0)	1 (10.0)
Non-union	0	0.0 (0.0; 0.3)							0 (−)	0 (−)
Pneumonia	20	1.6 (1.0; 2.4)	1 (5.0)	10 (50.0)	4 (20.0)		1 (5.0)	4 (20.0)	15 (75.0)	5 (25.0)
Postoperative neuropathic pain	9	0.7 (0.3; 1.3)		7 (77.8)	2 (22.2)				9 (100.0)	0 (0.0)
Pressure sores	4	0.3 (0.1; 0.8)	1 (25.0)	2 (50.0)	1 (25.0				4 (100.0)	0 (0.0)
Pulmonary embolism	21	1.7 (1.0; 2.5)		11 (52.4)	5 (23.8)	2 (9.5)		3 (14.3)	16 (76.2)	5 (23.8)
Superficial wound infection	21	1.7 (1.0; 2.5)	1 (4.8)	12 (57.1)	7 (33.3)	1 (4.8)			20 (95.2)	1 (4.8)
Systemic infection	11	0.9 (0.4; 1.5)		5 (45.5)	6 (54.5)				11 (100.0)	0 (0.0)
Urinary tract infection	31	2.4 (1.7; 3.5)	4 (12.9)	25 (80.6)	2 (6.5)				31 (100.0)	0 (0.0)
Wound dehiscence	32	2.5 (1.7; 3.5)	2 (6.3)	11 (34.4)	15 (46.9)	3 (9.4)	1 (3.1)		28 (87.5)	4 (12.5)
Any other postop AE	95	7.5 (6.1; 9.1)	14 (14.7)	52 (54.7)	22 (23.2)	6 (6.3)		1 (1.1)	88 (92.6)	7 (7.4)

^a^Number of patients with at least 1 AE. If a patient experienced multiple AEs under any AE class, the patient was only counted once.

^b^Estimated risk of developing at least 1 AE (calculated by dividing the number of patients experiencing at least 1 AE by the total number of patients).

^c^Confidence intervals for percentages were calculated using the exact method (Clopper Pearson method).

Note. All postoperative AEs considered with date of onset not later than 90 days after surgery to treat index lesion. If date of onset is after date of second surgery to treat index lesion the AE was not considered. The Adverse Event Severity system version 2 (SAVES V2) AE abstraction tool was used to record the morbidity data.

As shown in Table 6, most patients with intraoperative AEs had only 1 (84/87, 6.6%), while 91 patients out of 245 (37%) with postoperative AEs experienced more than 1.

**Table 6.** Number of Adverse Events per Subject (Patient Level).

	Total Number of Patients = 1267					
	Intraop			Post Op		
Number of Different Surgery-Related AEs per Subject, n (%)	n	Minor	Major	n	Minor	Major
0	1180 (93.1)	1187 (93.7)	1260 (99.4)	1022 (80.7)	1048 (82.7)	1233 (97.3)
1	84 (6.6)	77 (6.1)	7 (0.6)	154 (12.2)	134 (10.6)	28 (2.2)
2	2 (0.2)	2 (0.2)	0 (0.0)	55 (4.3)	52 (4.1)	5 (0.4)
3	1 (0.1)	1 (0.1)	0 (0.0)	17 (1.3)	18 (1.4)	0 (0.0)
4				8 (0.6)	5 (0.4)	1 (0.1)
5				2 (0.2)	3 (0.2)	0 (0.0)
6				5 (0.4)	4 (0.3)	0 (0.0)
7				2 (0.2)	1 (0.1)	0 (0.0)
8				0 (0.0)	0 (0.0)	0 (0.0)
9				1 (0.1)	1 (0.1)	0 (0.0)
10				0 (0.0)	0 (0.0)	0 (0.0)
11				1 (0.1)	1 (0.1)	0 (0.0)
12				0 (0.0)	0 (0.0)	0 (0.0)

Note. All intraoperative AEs which happen after the first documented surgery to treat index lesion were taken into consideration. If a second surgery was performed the subsequent reported intraoperative AEs were not taken into consideration. All postoperative AEs considered with date of onset not later than 90 days after surgery to treat index lesion. If date of onset is after date of second surgery to treat index lesion the AE was not considered. The Adverse Event Severity system version 2 (SAVES V2) AE abstraction tool was used to record the morbidity data.

The population characteristics (demographic variables, tumor variables, neurological variables, symptoms, surgical variables and previous treatments) were stratified in relation to the occurrence of intraoperative and postoperative AEs, as reported in Supplemental Materials (Tables I-X).

### Logistic Regression Models for the Occurrence of Adverse Events

Logistic regression models have been used to analyze the risk factors for the occurrence of AEs and the results are reported in Table 7. Only the amount of blood loss during surgery was shown to be a risk factor for intraoperative AEs, while several factors were shown to be associated with the occurrence of postoperative AEs: age, smoking status, ECOG Performance status (score 3-4), previous radiation therapy at the index target, duration of surgery, number of instrumented levels, number of surgical stages, simultaneous anterior and posterior approach, presence of metastases at other sites, and multiple spine metastases. A comprehensive overview of the variables considered (including factors that do not appear to have any impact) is provided in Supplemental Materials (Tables XI-XIII)

**Table 7.** Summary of Logistic Regression Model for Occurrence of AEs.

Category	Variable	Reference	Level	Estimate	St. Error	Lower	Upper	*P*-Value	OR	Lower	Upper
						Wald 95% Confidence Limits for Estimate				Wald 95% Confidence Limits for OR	
Intraoperative AES											
N = 1045 (76 subjects with intraoperative AE, 969 subjects without intraoperative AE)											
	Intercept			−4.692	0.907	−6.469	−2.914	<.0001			
Surgery	Duration of first surgery (hours)			0.136	0.070	−0.001	0.274	0.0510	1.146	0.999	1.315
	Estimated blood loss			0.001	0.000	0.001	0.001	<.0001	1.001	1.001	1.001
Postoperative AES											
N = 1044 (200 subjects with postoperative AE, 844 subjects without postoperative AE)											
	Intercept			−3.193	0.775	−4.713	−1.673	<.0001			
Demographics	Age at baseline (years)			0.022	0.008	0.007	0.037	0.0044	1.022	1.007	1.038
Neurological status	ECOG performance status	0	3/4	0.396	0.155	0.093	0.699	0.0105	2.445	1.263	4.733
Previous treatment	Did the patient receive radiation therapy to treat the index target PRIOR to inclusion?	No	Yes	0.372	0.106	0.165	0.580	0.0004	2.105	1.391	3.188
Surgery	Duration of first surgery (hours)			0.196	0.054	0.090	0.302	0.0003	1.216	1.094	1.352
	Estimated blood loss			0.000	0.000	0.000	0.001	0.0057	1.000	1.000	1.001
	Surgical approach	Anterior	Both (simultaneous)	0.935	0.410	0.131	1.738	0.0227	7.535	1.484	38.261
Any surgery-related AES											
N = 1044 (200 subjects with postoperative AE, 844 subjects without postoperative AE)											
	Intercept			−2.170	0.728	−3.598	−0.743	0.0029			
Demographics	Age at baseline (years)			0.023	0.007	0.009	0.037	0.0015	1.023	1.009	1.038
Neurological status	ECOG performance status	0	3/4	0.315	0.154	0.014	0.616	0.0404	2.058	1.103	3.838
Previous treatment	Did the patient receive radiation therapy to treat the index target PRIOR to inclusion?	No	Yes	0.318	0.101	0.119	0.516	0.0017	1.888	1.269	2.809
Surgery	Duration of first surgery (hours)			0.205	0.052	0.102	0.307	<.0001	1.227	1.108	1.359
	Estimated blood loss			0.000	0.000	0.000	0.001	<.0001	1.000	1.000	1.001
	Number of stages	1	2/3	1.060	0.267	0.536	1.583	<.0001	8.325	2.920	23.732
	Surgical approach	Anterior	Both (simultaneous)	0.806	0.399	0.025	1.587	0.0432	5.095	1.148	22.622
Tumor information	Location of metastatic spine tumor in L	No	Yes	−0.213	0.105	−0.419	−0.006	0.0436	0.654	0.433	0.988

### Impact of AEs on Survival

**Figure 3.**
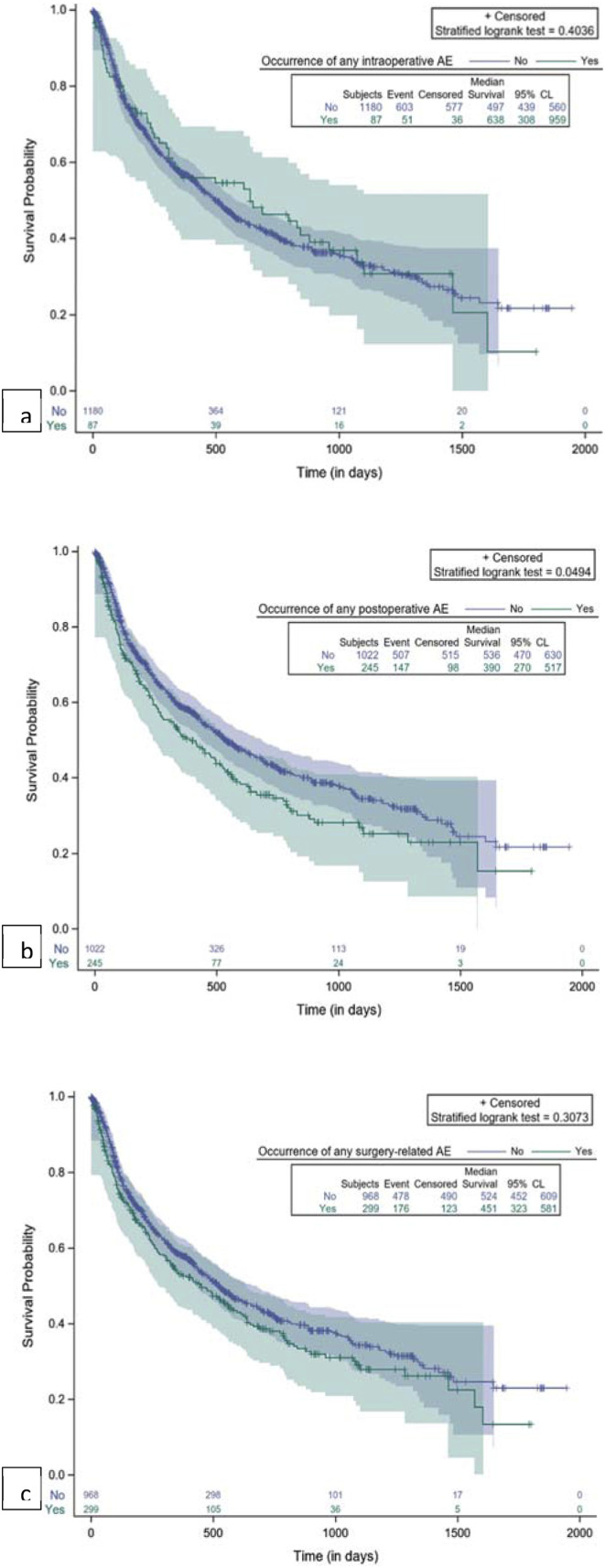
Kaplan-Meier plot for comparison of survival by occurrence of AE. (A) Any intraoperative AE; (B) Any postoperative AE; (C) Any surgery-related AE.

**Figure 4.**
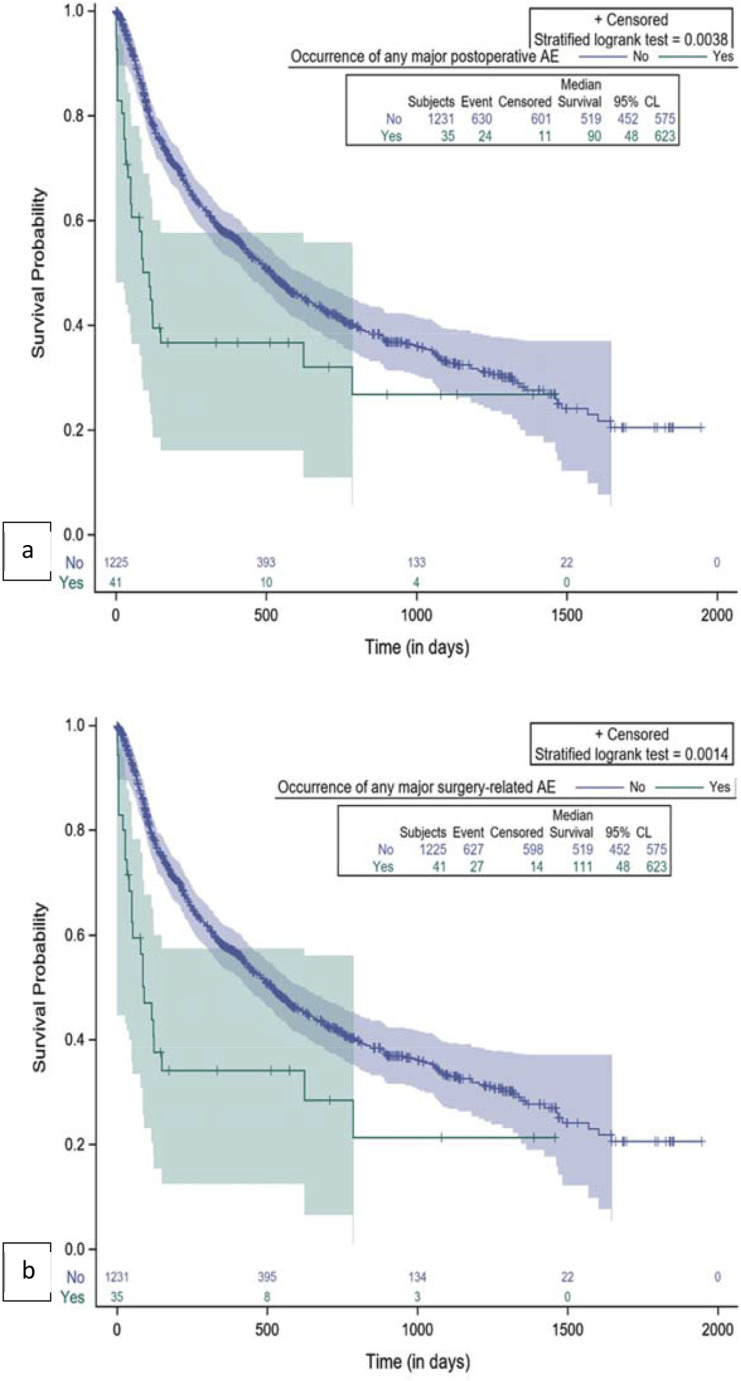
Kaplan-Meier plot for comparison of survival by occurrence of major AE. (A) Any major postoperative AE; (B) Any major surgery-related AE.

The impact of surgery-related AEs on patients’ survival was evaluated by Kaplan-Meier analysis and the plots are reported in Figures 3 and 4.

Any intraoperative AEs and major intraoperative AEs had no significant impact on survival (Figure 3A), while any postoperative AEs, and in particular major postoperative AEs, were significantly associated with shorter survival, as shown in Figure 3B and 4A. If we consider all surgery-related AEs, only major events had a significant association with survival time (Figure 4B. Patients without any major surgery-related AE had a median survival of 519 days (17.3 months) (95% CL: 452; 575). The occurrence of major surgery-related AEs was correlated with a significant decrease in survival (*P* = 0.0014), specifically a median survival of 111 days (3.7 months) (95% CL: 48; 623) (Figure 4B).

### Cox Regression Models for Survival

A multivariable analysis was performed to correlate several baseline variables to survival, using as variable the occurrence of intraoperative AEs or postoperative AEs or any surgery-related AEs (intra-op and post-op) (Table 8). Preliminary analysis reported in Supplemental Materials (Tables XIV-XVI) evaluated many variables, then only variables with *P*-value ≤0.06 were considered and the results of the post-hoc Cox-models are reported in Table 8. The multivariable analyses indicate that both intraoperative and postoperative AEs did not have a significant impact on survival, in the presence of other variables having a significant impact on the survival of patients suffering from spinal metastases. These variables are the presence of comorbidities (measured by Charlson Comorbidity Index, CCI), the ECOG Performance Status, the occurrence of previous surgery to the index lesion, neurological status (presence of high grade ESCC, presence of myelopathy), and the presence of visceral/brain metastases.

**Table 8.** Summary of Cox-Model for Survival With Occurrence of AEs as Input Variable.

Category	Variable	Reference	Level	Estimate	St. Error	*P*-Value	Hazard Ratio	Lower Limit	Upper Limit
								95% Confidence Limit for Hazard Ratio	
Intraoperative AES as input variable									
N = 1067 (event = 568, censored = 499)									
AE	Occurrence of any intraoperative AE	No	Yes	−0.107	0.155	0.4882	0.898	0.663	1.217
Demographics	Charlson comorbidity index (version 2)			0.107	0.029	0.0002	1.113	1.052	1.178
			3/4	0.758	0.169	<.0001	2.134	1.531	2.973
Prior treatment	Did the patient receive surgery to treat the index target PRIOR to inclusion?	No	Yes	−0.365	0.164	0.0261	0.694	0.503	0.958
Surgery	Procedure type	Palliative	Curettage	−0.502	0.113	<.0001	0.605	0.486	0.755
			En bloc	−0.760	0.208	0.0003	0.468	0.311	0.703
	Surgical indication//Neurological: High grade ESCC	No	Yes	0.258	0.086	0.0026	1.294	1.094	1.531
Tumor information	Visceral/brain metastases	No	Yes	0.586	0.087	<.0001	1.797	1.516	2.129
Postoperative AES as input variable									
N = 1067 (event = 568, censored = 499)									
AE	Occurrence of any postoperative AE	No	Yes	0.126	0.103	0.2209	1.135	0.927	1.389
Demographics	Charlson comorbidity index (version 2)			0.107	0.029	0.0002	1.113	1.051	1.178
Neurological status	ECOG performance status	0	1/2	0.412	0.156	0.0082	1.509	1.112	2.048
			3/4	0.702	0.169	<.0001	2.018	1.450	2.808
Surgery	Procedure type	Palliative	Curettage	−0.532	0.113	<.0001	0.587	0.471	0.732
			En bloc	−0.833	0.208	<.0001	0.435	0.289	0.654
	Surgical indication//Neurological: High grade ESCC	No	Yes	0.251	0.088	0.0041	1.285	1.083	1.526
	Surgical indication//Neurological: Myelopathy	No	Yes	0.213	0.095	0.0256	1.237	1.026	1.492
Tumor information	Visceral/brain metastases	No	Yes	0.588	0.087	<.0001	1.800	1.518	2.135
Any surgery-related AES as input variable									
N = 1067 (event = 568, censored = 499)									
AE	Occurrence of any surgery-related AE (intraop or postop)	No	Yes	0.023	0.097	0.8114	1.023	0.847	1.237
Demographics	Charlson comorbidity index (version 2)			0.112	0.029	0.0001	1.118	1.057	1.183
Neurological status	ECOG performance status	0	1/2	0.411	0.156	0.0083	1.508	1.111	2.045
			3/4	0.712	0.168	<.0001	2.038	1.466	2.835
Surgery	Procedure type	Palliative	Curettage	−0.525	0.112	<.0001	0.592	0.475	0.737
			En bloc	−0.810	0.208	<.0001	0.445	0.296	0.669
	Surgical indication//Neurological: High grade ESCC	No	Yes	0.251	0.086	0.0033	1.286	1.087	1.521
	Surgical indication//Neurological: Myelopathy	No	Yes	0.224	0.095	0.0190	1.251	1.037	1.508
Tumor information	Visceral/brain metastases	No	Yes	0.596	0.087	<.0001	1.814	1.530	2.151

Note. This post-hoc cox-model analysis takes only variables with *P*-value <= 0.06 from the results in Jan 2024.

### Impact of Surgery-Related AEs on Length of Hospital Stay

**Figure 5.**
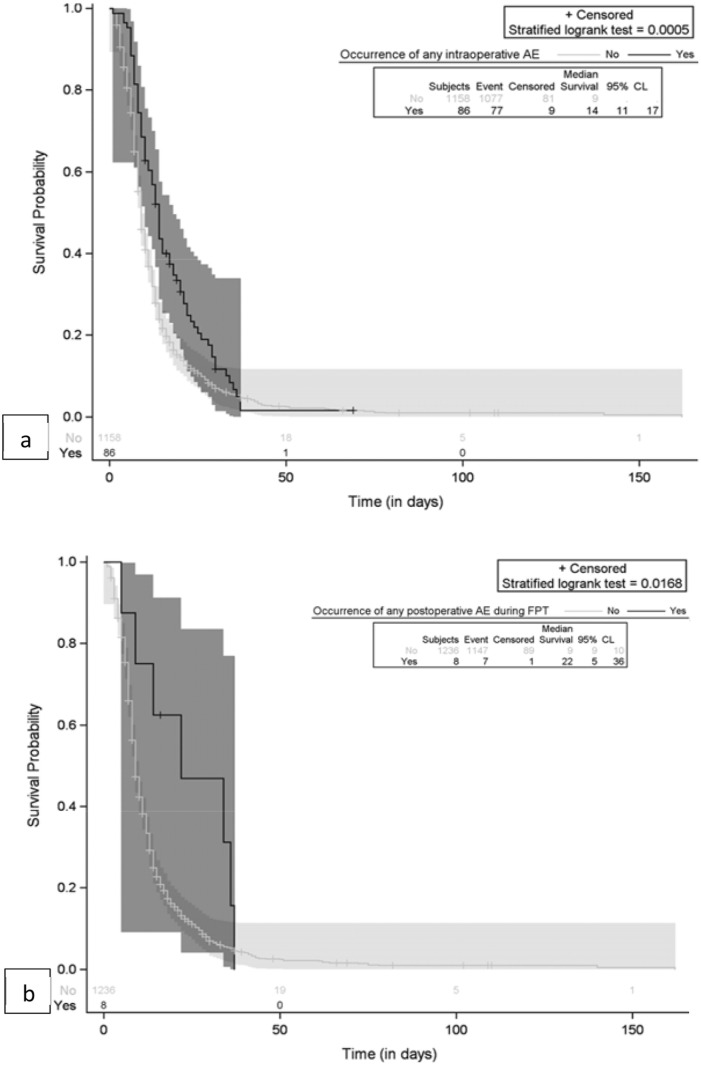
Kaplan-Meier plot for comparison length of hospital stay by occurrence of AE. (A) Any intraoperative AE; (B) Any postoperative AE (within the hospitalization time).

The impact of surgery-related AEs on patients’ length of stay (LOS) was evaluated by Kaplan-Meier analysis. As shown in Figure 5, both intraoperative and postoperative AEs during the hospitalization period were associated with longer LOS. The small number of AEs occurring after surgery and before patients’ discharge may have affected the results.

### Cox Regression Models for Length of Stay

A multivariable analysis was performed to correlate several baseline variables to the LOS, using as input variable the occurrence of intraoperative AEs or postoperative AEs or any surgery-related AEs (intraoperative and postoperative) (Table 9). The multivariable analyses confirmed the significant association of both intraoperative and postoperative complications on the length of stay. Other variables shown to be risk factors for a prolonged LOS were: age, neurological impairment (ASIA score A-C), ECOG Performance Status (score 3-4), potential spinal instability (SINS 7-12), number of instrumented levels, presence of metastases at other sites.

**Table 9.** Summary of Cox-Model for Time From Hospital Admission to Discharge Including AE as Input Variable.

Category	Variable	Reference	Level	Estimate	St. Error	*P*-Value	Hazard Ratio	Lower Limit	Upper Limit
								95% Confidence Limit for Hazard Ratio	
Intraoperative AES as input variable									
N = 967 (event = 893, censored = 74)									
AE	Occurrence of any intraoperative AE	No	Yes	−0.414	0.144	0.0040	0.661	0.499	0.876
Demographics	Age at baseline			−0.016	0.003	<.0001	0.984	0.978	0.989
Neurological status	ASIA impairment scale	D/E	A/B/C	−0.288	0.134	0.0318	0.750	0.577	0.975
			3/4	−0.322	0.130	0.0133	0.724	0.561	0.935
	Total SINS score	Stable: 0-6	Indeterminate: 7-12	−0.325	0.130	0.0121	0.722	0.560	0.931
Prior treatment	Did the patient receive systemic therapy to treat the metastatic disease PRIOR to inclusion?	No	Yes	0.295	0.100	0.0031	1.343	1.105	1.632
Surgery	Estimated blood loss during the procedure (mL)			−0.000	0.000	0.0220	1.000	1.000	1.000
	Number of instrumented levels			−0.059	0.017	0.0003	0.943	0.913	0.974
	Number of stages	1	2/3	−0.715	0.242	0.0031	0.489	0.304	0.786
			En bloc	−0.560	0.157	0.0004	0.571	0.420	0.777
	Surgical indication//Neurological: Myelopathy	No	Yes	−0.202	0.084	0.0162	0.817	0.692	0.963
	Does the patient have metastases at other site(s)?	No	Yes	−0.395	0.097	<.0001	0.674	0.556	0.815
	Location of metastatic spine tumor in C	No	Yes	0.233	0.111	0.0361	1.263	1.015	1.571
Postoperative AES (during hospitalization) as input variable									
N = 967 (event = 893, censored = 74)									
AE	Occurrence of any postoperative AE	No	Yes	−0.748	0.401	0.0617	0.473	0.216	1.037
Demographics	Age at baseline			−0.017	0.003	<.0001	0.983	0.978	0.989
Neurological status	ASIA impairment scale	D/E	A/B/C	−0.275	0.134	0.0403	0.760	0.584	0.988
	ECOG performance status		3/4	−0.293	0.130	0.0243	0.746	0.578	0.963
	Total SINS score	Stable: 0-6	Indeterminate: 7-12	−0.312	0.130	0.0166	0.732	0.567	0.945
Prior treatment	Did the patient receive systemic therapy to treat the metastatic disease PRIOR to inclusion?	No	Yes	0.303	0.100	0.0024	1.354	1.114	1.646
Surgery	Estimated blood loss during the procedure (mL)			−0.000	0.000	0.0147	1.000	1.000	1.000
	Number of instrumented levels			−0.061	0.017	0.0003	0.941	0.911	0.973
	Number of stages	1	2/3	−0.774	0.242	0.0014	0.461	0.287	0.741
			En bloc	−0.550	0.157	0.0005	0.577	0.424	0.785
	Surgical indication//Neurological: Myelopathy	No	Yes	−0.189	0.084	0.0252	0.828	0.702	0.977
	Surgical indication//Oncologic: RT resistant	No	Yes	0.386	0.103	0.0002	1.471	1.202	1.799
Tumor information	Does the patient have metastases at other site(s)?	No	Yes	−0.381	0.097	<.0001	0.683	0.565	0.827
	Location of metastatic spine tumor in C	No	Yes	0.211	0.111	0.0562	1.235	0.994	1.534

### Effect of Surgery and AEs on Patients’ Quality of Life

The effect of surgery on HRQOL was evaluated by patient-reported outcomes measures (PROMs). In particular, EQ-5D and SOSGOQ questionnaires were self-administered, and the results obtained at baseline, 2 months follow up and 6 months follow up were compared. Moreover, the impact of AEs on HRQOL was analyzed.

**Figure 6.**
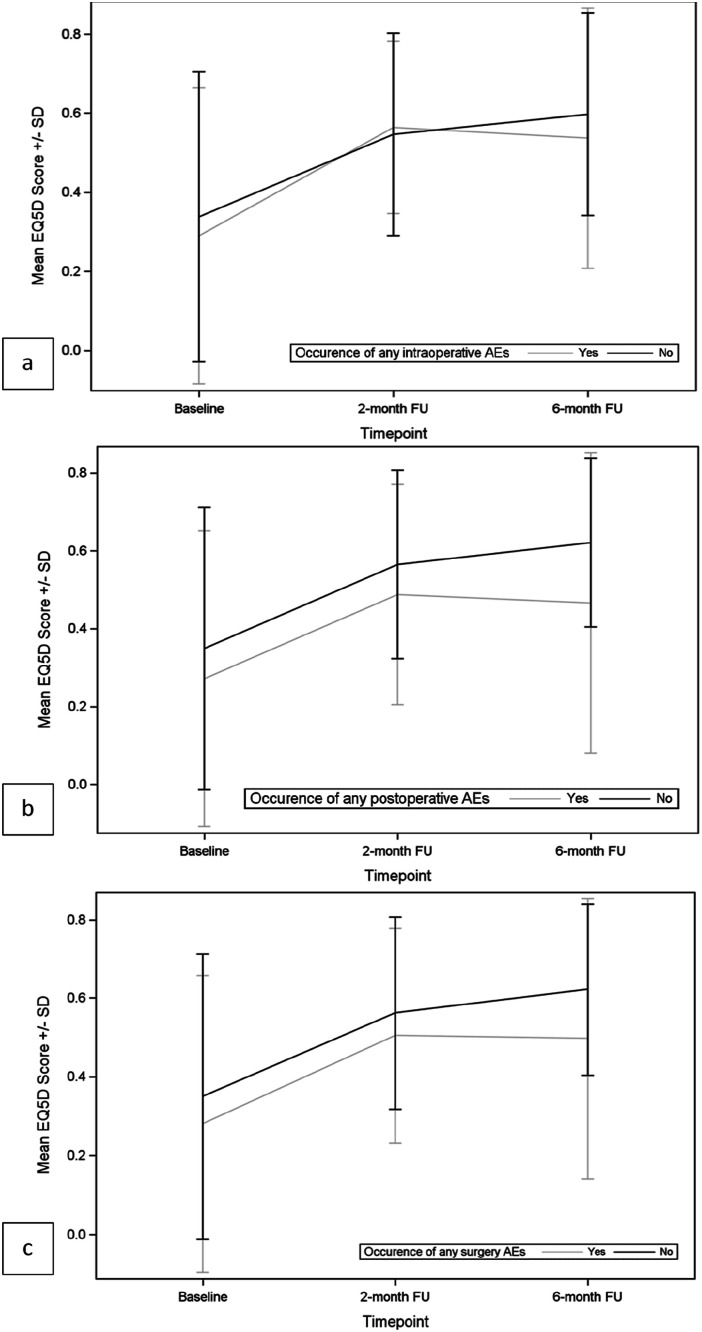
Mean EQ-5D scores by occurrence of AEs. (A) Intraoperative AEs; (B) postoperative AEs; (C) any surgery-related AEs.

**Figure 7.**
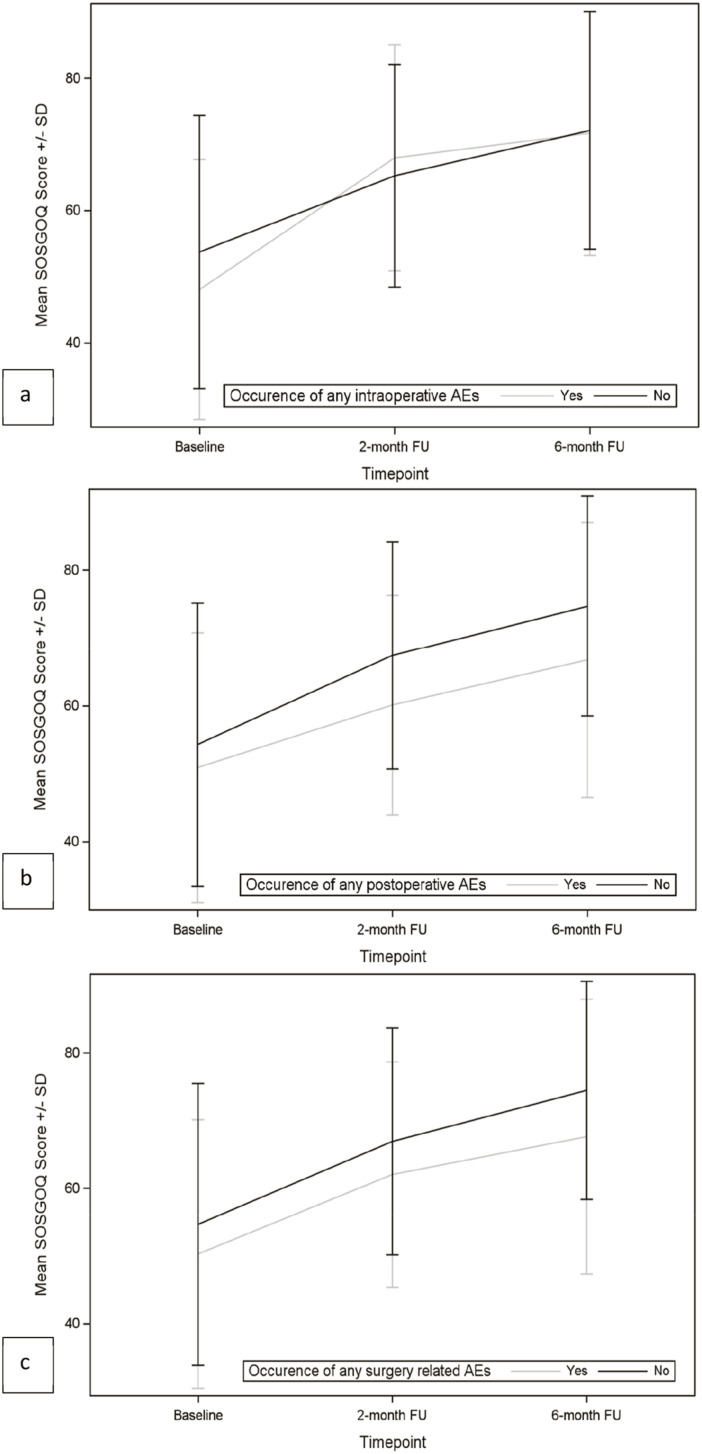
Mean SOSGOQ scores by occurrence of AEs. (A) Intraoperative AEs; (B) postoperative AEs; (C) any surgery-related AEs.

As shown in Figures 6 and 7 and in Tables 10 and 11, both EQ-5D score and SOSGOQ total score significantly improved immediately after the surgery, and the improvement increased during the follow up period. The improvement of EQ-5D and SOSGOQ values immediately after the surgery was recorded also in the presence of intraoperative and/or postoperative adverse events, even if no significant increase was recorded between 2 months and 6 months FU (Tables 10 and 11).

**Table 10.** Summary of EQ5D-3L Mean Values Including Standard Deviations and *P*-values by Occurrence of AEs.

Characteristic	Occurrence of Any Intraoperative AE		*P* Value
	No	Yes	
	N = 1180	N = 87	
EQ-5D-3L score at baseline			
Mean (SD)	0.34 (0.37)	0.29 (0.38)	0.2573
EQ-5D-3L score at 2 months			
Mean (SD)	0.55 (0.26)	0.56 (0.22)	0.7423
EQ-5D-3L score at 6 months			
Mean (SD)	0.60 (0.26)	0.54 (0.33)	0.1964
*P* values between timepoints			
Baseline vs 2-month FU	**<.0001**	**0.0008**	
Baseline vs 6-month FU	**<.0001**	**0.0010**	
2-month FU vs 6-month	**0.0093**	0.7243	

Characteristic	Occurrence of Any Postoperative AE		*P* Value
	No	Yes	
	N = 1022	N = 245	
EQ-5D-3L score at baseline			
Mean (SD)	0.35 (0.36)	0.27 (0.38)	**0.0049**
EQ-5D-3L score at 2 months			
Mean (SD)	0.56 (0.24)	0.49 (0.28)	**0.0193**
EQ-5D-3L score at 6 months			
Mean (SD)	0.62 (0.22)	0.47 (0.39)	**<.0001**
*P* Values between timepoints			
Baseline vs 2-month FU	**<.0001**	**<.0001**	
Baseline vs 6-month FU	**<.0001**	**0.0001**	
2-month FU vs 6-month	**0.0028**	0.6929	

Characteristic	Occurrence of Any Surgery-Related AE		*P* Value
	No	Yes	
	N = 968	N = 299	
EQ-5D-3L score at baseline			
Mean (SD)	0.35 (0.36)	0.28 (0.38)	**0.0068**
EQ-5D-3L score at 2 months			
Mean (SD)	0.56 (0.25)	0.50 (0.27)	0.0700
EQ-5D-3L score at 6 months			
Mean (SD)	0.62 (0.22)	0.50 (0.36)	**<.0001**
*P* Values between timepoints			
Baseline vs 2-month FU	**<.0001**	**<.0001**	
Baseline vs 6-month FU	**<.0001**	**<.0001**	
2-month FU vs 6-month	**0.0022**	0.8721	

*P* values are calculated by *t* test. Bold values represent *p* < 0.05 (statistically significant).

**Table 11.** Summary of SOSGOQ Total Score Mean Values Including Standard Deviations and P-values by Occurrence of AEs.

Characteristic	Occurrence of Any Intraoperative AE		*P* Value
	No	Yes	
	N = 1180	N = 87	
Total SOSGOQ score at baseline			
Mean (SD)	53.72 (20.71)	48.03 (19.74)	0.1528
Total SOSGOQ score at 2 months			
Mean (SD)	65.24 (16.91)	67.96 (17.18)	0.5298
Total SOSGOQ score at 6 months			
Mean (SD)	72.10 (18.02)	71.69 (18.56)	0.9389
*P* Values between timepoints			
Baseline vs 2-month FU	**<.0001**	**0.0012**	
Baseline vs 6-month FU	**<.0001**	**0.0007**	
2-month FU vs 6-month	**0.0009**	0.5731	

Characteristic	Occurrence of Any Postoperative AE		*P* Value
	No	Yes	
	N = 1022	N = 245	
Total SOSGOQ score at baseline			
Mean (SD)	54.28 (20.92)	50.89 (19.94)	0.1296
Total SOSGOQ score at 2 months			
Mean (SD)	67.40 (16.78)	60.09 (16.23)	**0.0097**
Total SOSGOQ score at 6 months			
Mean (SD)	74.66 (16.25)	66.73 (20.29)	**0.0138**
*P* Values between timepoints			
Baseline vs 2-month FU	**<.0001**	**0.0051**	
Baseline vs 6-month FU	**<.0001**	**<.0001**	
2-month FU vs 6-month	**0.0012**	0.0825	

Characteristic	Occurrence of Any Surgery-Related AE		*P* Value
	No	Yes	
	N = 968	N = 299	
Total SOSGOQ score at baseline			
Mean (SD)	54.64 (20.90)	50.27 (19.91)	**0.0461**
Total SOSGOQ score at 2 months			
Mean (SD)	66.90 (16.83)	62.01 (16.73)	0.0753
Total SOSGOQ score at 6 months			
Mean (SD)	74.45 (16.20)	67.61 (20.38)	**0.0315**
*P* Values between timepoints			
Baseline vs 2-month FU	**<.0001**	**0.0002**	
Baseline vs 6-month FU	**<.0001**	**<.0001**	
2-month FU vs 6-month	**0.0010**	0.1311	

*P* values are calculated by *t* test. Bold values represent *p* < 0.05 (statistically significant).

Comparing the mean values of EQ-5D and SOSGOQ scores between the 2 groups of patients with or without intraoperative AEs, we observed no significant differences at baseline and at follow-up timepoints (2 months or 6 months) (Tables 10 and 11). However, in the group of patients suffering postoperative or any surgery-related AEs, significantly different baseline values were detected for EQ-5D and SOSGOQ scores compared to the population without postoperative or any surgery-related AEs. The difference was confirmed also at follow-up timepoints (Tables 10 and 11).

Analyzing in detail the different domains of SOSGOQ (Supplemental Materials Table XVII), we observed a significant improvement for the Pain domain score, the Mental domain score and the Social domain score immediately after the surgery, and the improvement increased during the follow up period. The Physical function domain score improved starting from 2 months after surgery, while the Neurological domain score improved later, starting from 6 months FU. The occurrence of intraoperative AEs did not impair the trend of different SOSGOQ’s domain scores during time, while the occurrence of postoperative AEs significantly affected SOSGOQ’s domain scores during time, in particular the Neurological function domain and the Mental domain, where no significant improvements were registered after surgery and during follow up in the presence of postoperative adverse events.

### Analysis of Risk Factors for HRQOL Improvement by Mixed Models for Repeated Measures (MMRM)

We analyzed by MMRM the impact of different variables on HRQOL scores (EQ-5D total score and SOSGOQ total score) in the presence of AEs (Tables 12 and 13). As reported in Table 12, we observed that some baseline variables have an impact on EQ-5D total score regardless of intraoperative and postoperative AEs: presence of comorbidities (Charlson Comorbidity Index), current smoking status, neurological impairment (ASIA score A/B/C vs D/E), duration of surgery, and location of metastases at the sacral level. These variables can impair HRQOL, regardless of the occurrence of AEs. Local radiation therapy prior to surgery had an impact on EQ-5D score in combination with postoperative AEs, while anterior + posterior surgical approach had an impact on EQ-5D score in combination with intraoperative AEs. For SOSGOQ total score, we observed no significant effects of baseline variables (Table 13), but some results emerged from the analyses of specific SOSGOQ domains: for the Physical function domain score, the neurological impairment at baseline (ASIA score A/B/C vs D/E), the procedure type (curettage vs palliative) and the presence of visceral/brain metastases have a significant impact, regardless of the occurrence of AEs (intraoperative or postoperative) (Supplemental Materials Table XVIII); the neurological impairment at baseline (ASIA score A/B/C vs D/E) and the presence of visceral/brain metastases also had a significant impact on the Neurological domain score (Supplemental Materials Table XIX); for the Pain domain score, only the surgical approach (posterior vs anterior) had a significant impact, regardless of the occurrence of AEs (Supplemental Materials Table XX); for the Mental domain score, only the current state of smoking had a significant impact, regardless the occurrence of AEs (Supplemental Materials Table XXI); for the Social domain score, only the number of instrumented levels had a significant impact, regardless the occurrence of AEs (Supplemental Materials Table XXII).

**Table 12.** Summary of MMRM Model for Difference of EQ5D-3L Total Score and AEs as Input Variable.

Variable Group	Variable	Reference	Level	Intraoperative AE as Input Variable					Postoperative AE as Input Variable				
				Estimate	Standard Error	Lower Limit	Upper Limit	*P*-Value	Estimate	Standard Error	Lower Limit	Upper Limit	*P*-Value
	Intercept			0.765	0.113	0.543	0.988	0.000	0.761	0.111	0.543	0.979	0.000
AE	Occurrence of any intra/post AE	No	Yes	−0.015	0.044	−0.102	0.072	0.732	−0.124	0.030	−0.183	−0.065	0.000
Demographics	Age at baseline (years)			−0.001	0.001	−0.003	0.001	0.262	−0.001	0.001	−0.003	0.001	0.248
	Charlson comorbidity index			−0.019	0.009	−0.036	−0.002	0.029	−0.019	0.009	−0.036	−0.002	0.026
	Gender	Male	Female	−0.002	0.024	−0.048	0.044	0.930	0.003	0.023	−0.042	0.049	0.889
	Smoking status	No	Not assessed	0.011	0.030	−0.048	0.070	0.710	0.010	0.029	−0.048	0.067	0.734
			Yes - currently	−0.102	0.047	−0.194	−0.009	0.032	−0.090	0.046	−0.181	0.001	0.053
			Yes - previously	−0.011	0.031	−0.072	0.050	0.721	−0.005	0.030	−0.064	0.055	0.874
EQ5D3L	Baseline EQ5D3L score			−0.864	0.038	−0.939	−0.789	0.000	−0.876	0.038	−0.950	−0.803	0.000
Neurological status	ASIA impairment scale	D/E	A/B/C	−0.167	0.051	−0.268	−0.066	0.001	−0.167	0.050	−0.266	−0.068	0.001
	ECOG performance status	0	1/2	−0.047	0.033	−0.111	0.017	0.148	−0.041	0.032	−0.104	0.022	0.200
			3/4	0.007	0.048	−0.087	0.102	0.876	0.024	0.047	−0.069	0.117	0.610
	Total SINS score	Stable: 0-6	Indeterminate: 7-	−0.001	0.035	−0.070	0.068	0.976	−0.003	0.034	−0.070	0.064	0.934
			12										
			Unstable: 13-18	−0.009	0.041	−0.090	0.072	0.830	−0.002	0.040	−0.081	0.077	0.968
Previous treatment	Did the patient receive radiation therapy to treat the index target PRIOR to inclusion?	No	Yes	0.056	0.031	−0.005	0.117	0.071	0.075	0.031	0.015	0.135	0.015
	Did the patient receive surgery to treat the index target PRIOR to inclusion?	No	Yes	−0.065	0.047	−0.157	0.028	0.169	−0.064	0.046	−0.155	0.026	0.164
	Did the patient receive systemic therapy to treat the metastatic disease PRIOR to inclusion?	No	Yes	−0.024	0.033	−0.089	0.042	0.475	−0.026	0.033	−0.090	0.038	0.423
Surgery	Duration of first surgery (hours)			0.017	0.008	0.001	0.034	0.039	0.019	0.008	0.003	0.035	0.023
	Estimated blood loss			−0.000	0.000	−0.000	0.000	0.694	−0.000	0.000	−0.000	0.000	0.913
	Number of instrumented levels			−0.005	0.006	−0.016	0.006	0.333	−0.006	0.005	−0.017	0.004	0.237
	Number of stages	1	2/3	0.020	0.085	−0.148	0.187	0.816	0.053	0.083	−0.111	0.216	0.529
	Procedure type	Palliative procedure	Curettage	0.022	0.028	−0.033	0.077	0.434	0.029	0.027	−0.025	0.082	0.296
			En bloc	−0.024	0.044	−0.110	0.062	0.587	−0.004	0.043	−0.089	0.080	0.918
	Surgical approach	Anterior	Both	−0.256	0.117	−0.486	−0.026	0.029	−0.193	0.115	−0.420	0.033	0.094
			Posterior	−0.045	0.068	−0.178	0.088	0.508	−0.028	0.067	−0.159	0.103	0.675
	Surgical indication // High grade ESCC	No	Yes	−0.035	0.025	−0.085	0.014	0.160	−0.039	0.025	−0.087	0.010	0.115
Tumor information	Does the patient have metastases at other site(s)?	No	Yes	−0.049	0.032	−0.112	0.013	0.119	−0.044	0.031	−0.105	0.017	0.153
	Location of metastatic spine tumor in C	No	Yes	0.044	0.037	−0.028	0.116	0.233	0.038	0.036	−0.033	0.109	0.291
	Location of metastatic spine tumor in L	No	Yes	0.013	0.030	−0.046	0.072	0.666	0.001	0.030	−0.058	0.059	0.983
	Location of metastatic spine tumor in S	No	Yes	−0.098	0.044	−0.184	−0.012	0.026	−0.104	0.043	−0.188	−0.021	0.015
	Location of metastatic spine tumor in T	No	Yes	0.012	0.034	−0.054	0.078	0.714	0.006	0.033	−0.059	0.070	0.862
	Number of spine metastases			0.001	0.005	−0.009	0.011	0.875	0.001	0.005	−0.008	0.011	0.784
	Visceral/brain metastases	No	Yes	0.010	0.034	−0.057	0.077	0.778	0.015	0.033	−0.051	0.080	0.654
Time	Timepoint	2 Months	6 Months	0.035	0.015	0.005	0.065	0.022	0.033	0.015	0.003	0.063	0.030

**Table 13.** Summary of MMRM Model for Difference of SOSGOQ Total Score and AEs as Input Variable.

Variable Group	Variable	Reference	Level	Intraoperative AE as Input Variable					Postoperative AE as Input Variable				
				Estimate	Standard Error	Lower Limit	Upper Limit	*P*-Value	Estimate	Standard Error	Lower Limit	Upper Limit	*P*-Value
	Intercept			60.164	13.468	33.565	86.762	0.000	61.734	13.112	35.838	87.630	0.000
AE	Occurrence of any intraoperative AE	No	Yes	2.216	4.600	−6.878	11.311	0.631	−7.443	2.889	−13.152	−1.735	0.011
Demographics	Age at baseline (years)			0.092	0.116	−0.137	0.322	0.428	0.121	0.109	−0.095	0.337	0.271
	Charlson comorbidity index			0.064	0.859	−1.633	1.760	0.941	−0.051	0.839	−1.708	1.607	0.952
	Gender	Male	Female	−0.693	2.608	−5.846	4.460	0.791	−0.900	2.538	−5.916	4.115	0.723
	Smoking status	No	Not assessed	3.598	5.144	−6.566	13.761	0.485	3.346	5.007	−6.548	13.240	0.505
			Yes - currently	−0.933	5.117	−11.043	9.176	0.856	−1.097	4.997	−10.969	8.775	0.827
			Yes - previously	−4.507	2.879	−10.196	1.182	0.120	−4.101	2.811	−9.655	1.453	0.147
Neurological status	ASIA impairment scale	D/E	A/B/C	−4.824	6.374	−17.415	7.767	0.450	−6.033	6.198	−18.277	6.211	0.332
	ECOG performance status	0	1/2	−1.186	4.305	−9.693	7.321	0.783	−1.995	4.226	−10.345	6.355	0.638
			3/4	−0.281	5.559	−11.263	10.701	0.960	−0.184	5.426	−10.905	10.536	0.973
	Total SINS score	Stable: 0-6	Indeterminate: 7-	1.919	3.995	−5.975	9.814	0.632	0.958	3.831	−6.611	8.528	0.803
			12										
			Unstable: 13-18	7.215	4.254	−1.192	15.622	0.092	6.545	4.103	−1.563	14.652	0.113
Previous treatment	Did the patient receive radiation therapy to treat the index target PRIOR to inclusion?	No	Yes	0.744	2.877	−4.940	6.428	0.796	2.457	2.840	−3.154	8.068	0.388
	Did the patient receive surgery to treat the index target PRIOR to inclusion?	No	Yes	1.653	5.091	−8.400	11.707	0.746	2.346	4.945	−7.418	12.111	0.636
	Did the patient receive systemic therapy to treat the metastatic disease PRIOR to inclusion?	No	Yes	−0.221	3.128	−6.402	5.960	0.944	−0.494	3.052	−6.525	5.537	0.872
SOSGOQ	Baseline SOSGOQ total score			−0.725	0.073	−0.869	−0.581	0.000	−0.750	0.070	−0.890	−0.611	0.000
Surgery	Duration of first surgery (hours)			0.059	0.889	−1.697	1.816	0.947	0.130	0.862	−1.574	1.833	0.881
	Estimated blood loss			0.001	0.001	−0.002	0.004	0.633	0.002	0.001	−0.001	0.004	0.248
	Number of instrumented levels			−0.612	0.624	−1.846	0.622	0.329	−0.563	0.612	−1.772	0.646	0.359
	Number of stages	1	2/3	−6.343	9.148	−24.422	11.737	0.489	−3.414	8.851	−20.911	14.084	0.700
	Procedure type	Palliative procedure	Curettage	5.967	3.033	−0.025	11.960	0.051	5.828	2.967	−0.033	11.689	0.051
			En bloc	−8.029	4.860	−17.628	1.570	0.101	−7.431	4.694	−16.703	1.841	0.115
	Surgical approach	Anterior	Both	−19.157	12.421	−43.698	5.383	0.125	−13.607	12.313	−37.934	10.720	0.271
			Posterior	−14.249	9.122	−32.268	3.769	0.120	−12.569	8.882	−30.114	4.977	0.159
	Surgical indication // High grade ESCC	No	Yes	−1.086	2.759	−6.536	4.364	0.694	−1.388	2.664	−6.650	3.875	0.603
Tumor information	Does the patient have metastases at other site(s)?	No	Yes	−5.733	3.682	−13.006	1.541	0.122	−5.075	3.603	−12.194	2.043	0.161
	Location of metastatic spine tumor in C	No	Yes	−1.580	4.156	−9.793	6.634	0.704	−2.535	4.066	−10.573	5.502	0.534
	Location of metastatic spine tumor in L	No	Yes	0.551	3.356	−6.079	7.182	0.870	−0.808	3.320	−7.367	5.752	0.808
	Location of metastatic spine tumor in S	No	Yes	−1.929	5.107	−12.020	8.162	0.706	−2.249	4.974	−12.077	7.579	0.652
	Location of metastatic spine tumor in T	No	Yes	−1.936	3.602	−9.054	5.182	0.592	−2.803	3.525	−9.770	4.165	0.428
	Number of spine metastases			0.390	0.621	−0.837	1.617	0.531	0.479	0.605	−0.717	1.675	0.430
	Visceral/brain metastases	No	Yes	6.440	3.855	−1.178	14.057	0.097	6.394	3.750	−1.016	13.805	0.090
Time	Timepoint	2 Months	6 Months	4.927	1.589	1.772	8.082	0.003	5.038	1.594	1.872	8.204	0.002

## Discussion

In this large multicentric study, we analyzed data specific to AEs in a cohort of patients, who were predominantly potentially unstable, neurologically intact, with high grade epidural disease and good performance status, and underwent surgery for spinal metastases. Our focus was on the incidence and impact of both intraoperative and postoperative AEs. Importantly, we also examined the main clinical and procedural variables that could play a role in increasing the likelihood of AEs, their effect on quality of life and survival, and their influence on hospital length of stay.

### Adverse Events and Risk Factors

The study revealed that, among the 1267 patients who underwent surgery for spinal metastases, 6.9% experienced intraoperative AEs, with unintended durotomy being the most frequent complication (3.5%). Most of these intraoperative events (92%) were minor (severity grade ≤3), indicating that intraoperative complications were relatively well managed within this cohort. The incidence of durotomy observed in our patient cohort aligns with existing literature, which reports an intraoperative durotomy incidence ranging from 2% to 16%.^24,31-35^

In this context, it is important to emphasize that patients with spinal metastases who undergo surgery for spinal metastases that were previously radiated show a significantly increased risk of dural tears^36^ due to the presence of epidural fibrosis, adhesions, and dura thinning. In our cohort 24% of patients had a history of prior radiation at the index target.

Postoperative AEs were more common (19.3%) than intraoperative AEs and primarily consisted of deep wound infections, wound dehiscence, and urinary infections. Notably, patients with postoperative events were more likely to experience multiple AEs (37% of those with postoperative AEs). This finding is also consistent with the literature; in spinal surgery for tumors and metastases, wound infection is 1 of the most reported complications, with an incidence ranging from 1.5% to 30%.^37-41^ Such infections often lead to multiple reoperations, increased costs, prolonged disability, and, occasionally, sepsis or even death.^42^ The clinical spectrum of wound infections is broad: they may be treatable with antibiotics alone, may require multiple wound debridement and revision surgeries, or may present with persistent dehiscence leading to sepsis and/or death.^37-41^ However, in our patient cohort, only 7/33 patients (21.2%) had a severe wound infection. As previously reported, cancer patients may frequently receive postoperative spinal radiation, and are potentially undergoing chemotherapy, all of which are risk factors for wound healing complications.^43,44^ In addition to radiotherapy and systemic therapies, other risk factors for wound infection include prior spinal surgeries, complex plastic reconstructions, an increasing number of comorbidities (in our study cohort, the Charlson Comorbidity Index was 6.44, range, 2-15), prolonged hospital stays, underlying neurological disorders, smoking, drug or alcohol abuse, underlying cardiac disorders (other than hypertension), diabetes, and obesity.^45^

Through logistic regression models our study also found that intraoperative blood loss is a significant risk factor for intraoperative AEs, whereas several factors are associated with postoperative AEs, including age, smoking habits, ECOG performance status (score 3-4), prior radiation therapy, surgery duration, number of instrumented levels, multiple surgical stages, and simultaneous anterior and posterior approaches. These findings underscore the influence of both patient-specific clinical conditions and procedural complexity on the likelihood of postoperative complications. The identification of these risk factors is crucial as it can guide preoperative planning, help healthcare providers anticipate potential issues, and implement targeted strategies to improve patient outcomes and reduce complications.

### Impact of AEs on Survival, Length of Stay and HRQOL

Concerning the impact of surgery-related AEs on survival, it was shown that intraoperative AEs, including major ones, do not significantly affect survival. However, as reported for other clinical studies, in this study postoperative AEs, especially major events, are significantly associated with reduced survival.^22,46^ In our study, patients without surgery-related AEs have a median survival of 524 days, while patients with major AEs show a survival reduction to 111 days. However, multivariable analyses suggested that the negative impact of postoperative AEs on survival could be associated with the presence of other factors having a significant impact on the survival of patients suffering from vertebral metastases: the presence of comorbidities, the poor ECOG Performance Status, the occurrence of a previous surgery to treat the index lesion (indicating presence of a recurrence), the neurological status (presence of high grade ESCC, presence of myelopathy), the presence of visceral/brain metastases. All these factors can create a frailty condition that (i) increases the risk of complications and (ii) impairs life expectancy. Moreover, the occurrence of complications can delay or prevent the possibility of adjuvant therapies (radiotherapy and systemic therapy), with consequent impact on survival.

Moreover, both intraoperative and postoperative AEs are also significantly associated with prolonged LOS. This effect on LOS is further confirmed by Cox regression models, where variables such as advanced age, spinal instability, neurological impairment (ASIA score A-C), and high ECOG performance status scores are also significant predictors of extended LOS. These results indicate that surgery-related complications can lead to a higher healthcare burden and extended hospitalization, which may have implications for patients’ recovery and quality of life and for healthcare costs.

The findings of this study also demonstrate that surgery significantly improves HRQOL, as measured by EQ-5D and SOSGOQ scores, with benefits becoming evident immediately after the procedure and continuing to increase over time. These results align with the existing literature, which consistently highlights the positive impact of surgical interventions on HRQOL for patients with complex conditions, such as metastatic disease or musculoskeletal tumors.^47-49^

However, the study also highlights a notable distinction between the impact of intraoperative AEs and postoperative AEs on patients’ HRQOL. Specifically, no significant differences were observed in the EQ-5D and SOSGOQ scores between patients with or without intraoperative AEs at baseline or at follow-up timepoints. This suggests that intraoperative AEs, at least within this cohort, may not have a detectable immediate or long-term effect on these HRQOL measures. Potential explanations include effective intraoperative management or a generally low severity of these events.

Conversely, patients who experienced postoperative AEs demonstrated significantly lower baseline EQ-5D and SOSGOQ scores compared to those without such events. This disparity persisted across follow-up timepoints, indicating a likely pre-existing vulnerability to postoperative AEs and a prolonged negative impact of these preoperative characteristics and the associated AEs on recovery trajectories. In other words, these patients not only started from a worse health status but were also more likely to experience a less favorable recovery process, highlighting the compounded burden of postoperative complications.^50^ From a clinical perspective, these findings underscore the importance of preoperative risk stratification to identify vulnerable patients who may require tailored care to mitigate the risks of postoperative AEs.

Analyzing the specific domains of SOSGOQ provides a deeper understanding of these dynamics. The Pain domain, along with the Mental and Social domains, showed significant improvements immediately after surgery. These findings are consistent with reports by other authors^51,52^ who documented rapid improvements in pain and psychological well-being following surgical interventions for spinal metastases. In contrast, the Physical Function domain improved starting at 2 months post-surgery, while the Neurological domain only began to improve at 6 months, suggesting that some domains require longer recovery periods. The delayed improvement in the Physical Function domain reflects the time required for musculoskeletal healing and the gradual adaptation to physical rehabilitation.^53^ The physical recovery process often involves wound healing, resolution of inflammation, and regaining strength and mobility, which take weeks to months.^54^ The even later improvement in the Neurological domain, observed at 6 months post-surgery, suggests that nerve function recovery is a longer and more complex process. Neurological improvements are dependent on factors such as the duration and degree of preoperative nerve compression, the extent of decompression achieved during surgery, and the inherent regenerative capacity of the nervous system, which is often slow.^55^ In cases where preoperative neurological deficits were severe, recovery may require extended periods for neural plasticity and regeneration to occur.^56^

Using Mixed Models for Repeated Measures (MMRM), several baseline factors emerged as significant predictors of HRQOL improvement, independent of AEs. These included comorbidities, smoking status, baseline neurological impairment, longer surgery duration, and sacral metastases. Patients with more comorbidities or who smoked demonstrated less robust improvements, reflecting the broader detrimental effects of systemic health conditions and lifestyle factors on recovery. Longer surgeries and sacral metastases likely represent more complex clinical cases, which are inherently associated with more demanding surgeries and greater challenges in achieving optimal outcomes. Additionally, baseline neurological impairment was a strong determinant, as reflected in the burden on patients’ quality of life and prolonged recovery periods. These findings align with reports from the AO Spine Knowledge Forum Tumor,^57^ which similarly highlighted the negative impact of comorbidities and smoking on surgical outcomes in cancer patients.

### Highlights and Limitations

The results of this study provide a comprehensive analysis of AEs associated with spinal metastases surgery, offering important insights into the incidence, nature, and impact of both intraoperative and postoperative complications. The prospective collection of a large cohort of cases in different clinical centers around the world increases the variability and generalizability of results. However, although the broad sample increases the potential for generalization, it does not inherently eliminate biases that may arise from the study’s design and the intrinsic limitations of the registry. The main limitation of this study is the possibility of different recordings of AEs in clinical centers, the presence of missing data and “lost to follow up” that may cause an underestimation of the incidence of AEs. Moreover, although our regression models identified certain variables associated with an increased risk of AEs, we cannot establish direct causation. The association between specific variables, such as advanced age or comorbid conditions, and AEs does not exclude the possibility that other factors not considered in our study may contribute to the observed outcomes.

Despite these limits, this study highlights some relevant findings for the management of patients with spinal metastases. The surgical treatment, which is indicated to restore the neurological function, treat spinal instability and/or pathological fracture and improve quality of life, results in a high rate of adverse events, in particular postoperative AEs, that have a pronounced effect on patient recovery, quality of life, and survival. The findings underscore that postoperative AEs, especially severe ones, significantly reduce survival rates and adversely impact HRQOL, particularly in domains such as neurological function and mental health. However, our analysis also reveals that some parameters indicating a frailty status (comorbidities, poor ECOG Performance Status, presence of recurrences, impaired neurological status, presence of visceral/brain metastases) are associated to the occurrence of surgery-related AEs, suggesting that these factors can contribute to the effect of AEs on survival and quality of life.

This finding is relevant as it underscores the importance of an accurate preoperative assessment to identify vulnerable patients who are more likely to experience surgical complications with consequent impact on recovery, quality of life, and survival.

Strategies to minimize the incidence and severity of postoperative complications, such as precise surgical planning, effective wound management, and timely adjuvant therapies, are crucial. Additionally, integrating tailored rehabilitation programs and addressing mental health challenges in postoperative care can further enhance outcomes and recovery trajectories.

## Conclusion

This large, multicentric study highlights the delicate balance required in treating complex conditions like spinal metastases, where the benefits of surgery must be weighed against the risks of complications. By identifying key risk factors for complications and their implications, this research underscores the importance of preoperative risk stratification to identify vulnerable patients who may require tailored perioperative care to mitigate the risks of postoperative AEs and their negative impact on survival and quality of life.

## Supplemental Material

Supplemental Material - Evaluation of Adverse Events and the Impact on Health-Related Outcomes in Patients Undergoing Surgery for Metastatic Spine Tumors: Analysis of the Metastatic Tumor Research and Outcomes Network (MTRON) Registry Dataset Supplemental Material for Evaluation of Adverse Events and the Impact on Health-Related Outcomes in Patients Undergoing Surgery for Metastatic Spine Tumors: Analysis of the Metastatic Tumor Research and Outcomes Network (MTRON) Registry Dataset by Giovanni Barbanti Brodano, Cristiana Griffoni, Francesca Salamanna, Luigi Emanuele Noli, Annalisa Monetta, Alessandro Luzzati, Alexander C. Disch, Aron Lazary, Ori Barzilai, Ilya Laufer, Ziya L. Gokaslan, Michael G. Fehlings, Jorrit-Jan Verlaan, Dean Chou, Laurence D. Rhines, John H. Shin, William G. J. Teixeira, Daniel M. Sciubba^16^, Chetan Bettegowda, Raphaële Charest-Morin, Stefano Boriani, Tony Goldschlager, Michael H. Weber, Michelle J. Clarke, John E. O’Toole, Cordula Netzer, C. Rory Goodwin, Addisu Mesfin, Praveen V. Mummaneni, Nicolas Dea, Jeremy J. Reynolds, Arjun Sahgal, Charles G. Fisher, Alessandro Gasbarrini, and AO Spine Knowledge Forum Tumor in Global Spine Journal.

## Data Availability

Data are collected in a secured RedCap database under the supervision of AO Network Clinical Research and the AO Innovation Translation Center, Clinical Evidence.*
