# Supplementary material for: Evaluation of Adverse Events and the Impact on Health-Related Outcomes in Patients Undergoing Surgery for Metastatic Spine Tumors: Analysis of the Metastatic Tumor Research and Outcomes Network (MTRON) Registry Dataset
Source: Global Spine J. 2025 Jun 6;16(1):410–33. doi: 10.1177/21925682251347247 (PMC12145413; doi:10.1177/21925682251347247)
Supplement: Supplemental Material - Evaluation of Adverse Events and the Impact on Health-Related Outcomes in Patients Undergoing Surgery for Metastatic Spine Tumors: Analysis of the Metastatic Tumor Research and Outcomes Network (MTRON) Registry Dataset [file sj-pdf-1-gsj-10.1177_21925682251347247.pdf]

Table I. Summary of demographic variables by occurrence of intraoperative AE

	Occurrence of any intraoperative AE		
**Baseline characteristic**	**No N = 1180**	**Yes N = 87**	**Total N = 1267**
4.1 Gender, n (%)	1180	87	1267
Female	529 (44.83)	37 (42.53)	566 (44.67)
Male	651 (55.17)	50 (57.47)	701 (55.33)
Age at baseline (years)			
n	1180	87	1267
Mean (sd)	61.97 (12.15)	62.89 (12.28)	62.03 (12.15)
Median (Q1; Q3)	63.00 (55.00; 71.00)	65.00 (55.00; 70.00)	63.00 (55.00; 71.00)
Min; Max	17.00; 89.00	25.00; 87.00	17.00; 89.00
Charlson Comorbidity Index			
n	1177	87	1264
Mean (sd)	6.40 (1.38)	6.89 (1.66)	6.44 (1.41)
Median (Q1; Q3)	6.00 (6.00; 7.00)	6.00 (6.00; 8.00)	6.00 (6.00; 7.00)
Min; Max	2.00; 15.00	2.00; 12.00	2.00; 15.00
Was smoking status assessed?, n (%)	1179	87	1266
No	289 (24.51)	26 (29.89)	315 (24.88)
Yes	890 (75.49)	61 (70.11)	951 (75.12)
5.1 Does the patient smoke or use chewing tobacco?, n (%)	889	61	950
No	551 (61.98)	42 (68.85)	593 (62.42)
Yes - currently	96 (10.80)	4 (6.56)	100 (10.53)
Yes - previously	242 (27.22)	15 (24.59)	257 (27.05)

Table II. Summary of demographic variables by occurrence of postoperative AE

	Occurrence of any postoperative AE		
**Baseline characteristic**	**No**<br>**N = 1022**	**Yes**<br>**N = 245**	**Total**<br>**N = 1267**
4.1 Gender, n (%)	1022	245	1267
Female	465 (45.50)	101 (41.22)	566 (44.67)
Male	557 (54.50)	144 (58.78)	701 (55.33)
Age at baseline (years)			
n	1022	245	1267
Mean (sd)	61.58 (12.16)	63.91 (11.98)	62.03 (12.15)
Median (Q1; Q3)	63.00 (55.00; 70.00)	65.00 (57.00; 72.00)	63.00 (55.00; 71.00)
Min; Max	17.00; 86.00	22.00; 89.00	17.00; 89.00
Charlson Comorbidity Index			
n	1020	244	1264
Mean (sd)	6.39 (1.38)	6.63 (1.51)	6.44 (1.41)
Median (Q1; Q3)	6.00 (6.00; 7.00)	6.00 (6.00; 7.00)	6.00 (6.00; 7.00)
Min; Max	2.00; 15.00	2.00; 13.00	2.00; 15.00
Was smoking status assessed?, n (%)	1021	245	1266
No	268 (26.25)	47 (19.18)	315 (24.88)
Yes	753 (73.75)	198 (80.82)	951 (75.12)
5.1 Does the patient smoke or use chewing tobacco?, n (%)	752	198	950
No	482 (64.10)	111 (56.06)	593 (62.42)
Yes - currently	71 (9.44)	29 (14.65)	100 (10.53)
Yes - previously	199 (26.46)	58 (29.29)	257 (27.05)

Table III. Summary of tumor variables by occurrence of intraoperative AE

	Occurrence of any intraoperative AE		
**Baseline characteristic**	**No<br>N = 1180**	**Yes<br>N = 87**	**Total<br>N = 1267**
Site of the primary tumor, n (%)	1179	87	1266
Breast	208 (17.6)	12 (13.8)	220 (17.4)
Lungs	194 (16.5)	8 (9.2)	202 (16.0)
Prostate	122 (10.3)	9 (10.3)	131 (10.3)
Kidney	165 (14.0)	24 (27.6)	189 (14.9)
Other	490 (41.6)	34 (39.1)	524 (41.4)
3.1 Does the patient have metastases at other site(s)?, n (%)	1180	87	1267
No	499 (42.3)	45 (51.7)	544 (42.9)
Yes	681 (57.7)	42 (48.3)	723 (57.1)
Location of metastatic spine tumor in C, n (%)	1180	87	1267
No	937 (79.4)	72 (82.8)	1009 (79.6)
Yes	243 (20.6)	15 (17.2)	258 (20.4)
Location of metastatic spine tumor in T, n (%)	1180	87	1267
No	346 (29.3)	31 (35.6)	377 (29.8)
Yes	834 (70.7)	56 (64.4)	890 (70.2)
Location of metastatic spine tumor in L, n (%)	1180	87	1267
No	653 (55.3)	46 (52.9)	699 (55.2)
Yes	527 (44.7)	41 (47.1)	568 (44.8)
Location of metastatic spine tumor in S, n (%)	1180	87	1267
No	1056 (89.5)	72 (82.8)	1128 (89.0)
Yes	124 (10.5)	15 (17.2)	139 (11.0)
Visceral/brain metastases, n (%)	1180	87	1267
No	743 (63.0)	57 (65.5)	800 (63.1)
Yes	437 (37.0)	30 (34.5)	467 (36.9)
Number of spine metastases			
n	1180	87	1267
Mean (sd)	3.3 (3.8)	3.1 (3.1)	3.3 (3.7)
Median (Q1; Q3)	2.0 (1.0; 4.0)	2.0 (1.0; 3.0)	2.0 (1.0; 4.0)
Min; Max	1.0; 31.0	1.0; 18.0	1.0; 31.0

Table IV. Summary of tumor variables by occurrence of postoperative AE

	Occurrence of any postoperative AE		
**Baseline characteristic**	**No<br>N = 1022**	**Yes<br>N = 245**	**Total<br>N = 1267**
Site of the primary tumor, n (%)	1021	245	1266
Breast	193 (18.9)	27 (11.0)	220 (17.4)
Lungs	161 (15.8)	41 (16.7)	202 (16.0)
Prostate	106 (10.4)	25 (10.2)	131 (10.3)
Kidney	138 (13.5)	51 (20.8)	189 (14.9)
Other	423 (41.4)	101 (41.2)	524 (41.4)
3.1 Does the patient have metastases at other site(s)?, n (%)	1022	245	1267
No	452 (44.2)	92 (37.6)	544 (42.9)
Yes	570 (55.8)	153 (62.4)	723 (57.1)
Location of metastatic spine tumor in C, n (%)	1022	245	1267
No	812 (79.5)	197 (80.4)	1009 (79.6)
Yes	210 (20.5)	48 (19.6)	258 (20.4)
Location of metastatic spine tumor in T, n (%)	1022	245	1267
No	308 (30.1)	69 (28.2)	377 (29.8)
Yes	714 (69.9)	176 (71.8)	890 (70.2)
Location of metastatic spine tumor in L, n (%)	1022	245	1267
No	554 (54.2)	145 (59.2)	699 (55.2)
Yes	468 (45.8)	100 (40.8)	568 (44.8)
Location of metastatic spine tumor in S, n (%)	1022	245	1267
No	911 (89.1)	217 (88.6)	1128 (89.0)
Yes	111 (10.9)	28 (11.4)	139 (11.0)
Visceral/brain metastases, n (%)	1022	245	1267
No	661 (64.7)	139 (56.7)	800 (63.1)
Yes	361 (35.3)	106 (43.3)	467 (36.9)
Number of spine metastases			
n	1022	245	1267
Mean (sd)	3.2 (3.5)	3.5 (4.5)	3.3 (3.7)
Median (Q1; Q3)	2.0 (1.0; 4.0)	2.0 (1.0; 4.0)	2.0 (1.0; 4.0)
Min; Max	1.0; 30.0	1.0; 31.0	1.0; 31.0

Table V. Summary of neurological variables / symptoms by occurrence of intraoperative AE

	Occurrence of any intraoperative AE		
**Baseline characteristic**	**No N = 1180**	**Yes N = 87**	**Total N = 1267**
Has ASIA score been assessed?, n (%)	1180	87	1267
No	61 (5.17)	1 (1.15)	62 (4.89)
Yes	1119 (94.83)	86 (98.85)	1205 (95.11)
ASIA Impairment Scale, n (%)	1114	86	1200
A/B/C	110 (9.87)	7 (8.14)	117 (9.75)
D/E	1004 (90.13)	79 (91.86)	1083 (90.25)
Have symptoms been assessed?, n (%)	1180	87	1267
No	3 (0.25)	0 (0.00)	3 (0.24)
Yes	1177 (99.75)	87 (100.00)	1264 (99.76)
Epidural Compression for most severe compression of index target?, n (%)	1126	84	1210
Low: 0-1c	447 (39.70)	27 (32.14)	474 (39.17)
High: 2-3	679 (60.30)	57 (67.86)	736 (60.83)
ECOG Performance Status, n (%)	1170	87	1257
0	152 (12.99)	11 (12.64)	163 (12.97)
1/2	742 (63.42)	53 (60.92)	795 (63.25)
3/4	276 (23.59)	23 (26.44)	299 (23.79)
5	0 (0.00)	0 (0.00)	0 (0.00)
Has SINS score been assessed?, n (%)	1180	87	1267
No	24 (2.03)	1 (1.15)	25 (1.97)
Yes	1156 (97.97)	86 (98.85)	1242 (98.03)
Total SINS score, n (%)	1138	86	1224
Stable: 0-6	116 (10.19)	19 (22.09)	135 (11.03)
Indeterminate: 7-12	761 (66.87)	49 (56.98)	810 (66.18)
Unstable: 13-18	261 (22.93)	18 (20.93)	279 (22.79)

Table VI. Summary of neurological variables / symptoms by occurrence of postoperative AE

	Occurrence of any postoperative AE		
**Baseline characteristic**	**No<br>N = 1022**	**Yes<br>N = 245**	**Total<br>N = 1267**
Has ASIA score been assessed?, n (%)	1022	245	1267
No	58 (5.68)	4 (1.63)	62 (4.89)
Yes	964 (94.32)	241 (98.37)	1205 (95.11)
ASIA Impairment Scale, n (%)	959	241	1200
A/B/C	88 (9.18)	29 (12.03)	117 (9.75)
D/E	871 (90.82)	212 (87.97)	1083 (90.25)
Have symptoms been assessed?, n (%)	1022	245	1267
No	3 (0.29)	0 (0.00)	3 (0.24)
Yes	1019 (99.71)	245 (100.00)	1264 (99.76)
Epidural Compression for most severe compression of index target?, n (%)	977	233	1210
Low: 0-1c	404 (41.35)	70 (30.04)	474 (39.17)
High: 2-3	573 (58.65)	163 (69.96)	736 (60.83)
ECOG Performance Status, n (%)	1014	243	1257
0	142 (14.00)	21 (8.64)	163 (12.97)
1/2	643 (63.41)	152 (62.55)	795 (63.25)
3/4	229 (22.58)	70 (28.81)	299 (23.79)
5	0 (0.00)	0 (0.00)	0 (0.00)
Has SINS score been assessed?, n (%)	1022	245	1267
No	21 (2.05)	4 (1.63)	25 (1.97)
Yes	1001 (97.95)	241 (98.37)	1242 (98.03)
Total SINS score, n (%)	990	234	1224
Stable: 0-6	110 (11.11)	25 (10.68)	135 (11.03)
Indeterminate: 7-12	659 (66.57)	151 (64.53)	810 (66.18)
Unstable: 13-18	221 (22.32)	58 (24.79)	279 (22.79)

Table VII. Summary of surgery variables by occurrence of intraoperative AE

Baseline characteristic	Occurrence of any intraoperative AE<br>No<br>N = 1180	Yes<br>N = 87	Total<br>N = 1267
1.9 What was the surgical approach within this first surgical stage?, n (%)	1178	87	1265
Anterior	40 (3.40)	5 (5.75)	45 (3.56)
Posterior	1119 (94.99)	80 (91.95)	1199 (94.78)
Both (simultaneous)	19 (1.61)	2 (2.30)	21 (1.66)
Number of stages, n (%)	1179	87	1266
1	1156 (98.05)	80 (91.95)	1236 (97.63)
2/3	23 (1.95)	7 (8.05)	30 (2.37)
1.5.1 Estimated blood loss during the procedure [ml]			
n	1086	78	1164
Mean (sd)	611.90 (580.62)	1396.73 (1468.33)	664.49 (704.13)
Median (Q1; Q3)	500.00 (230.00; 800.00)	1000.00 (500.00; 1800.00)	500.00 (250.00; 800.00)
Min; Max	0.00; 6000.00	10.00; 7500.00	0.00; 7500.00
Duration of first surgery (hours)			
n	1167	87	1254
Mean (sd)	3.37 (1.80)	4.20 (2.37)	3.43 (1.86)
Median (Q1; Q3)	3.00 (2.17; 4.05)	3.75 (2.50; 5.08)	3.05 (2.20; 4.08)
Min; Max	0.17; 12.03	0.32; 13.13	0.17; 13.13
Number of instrumented levels			
n	1180	87	1267
Mean (sd)	4.78 (2.76)	5.07 (3.03)	4.80 (2.78)
Median (Q1; Q3)	5.00 (3.00; 6.00)	5.00 (3.00; 7.00)	5.00 (3.00; 6.00)
Min; Max	0.00; 20.00	0.00; 15.00	0.00; 20.00
Surgical Indication // Neurological: Functional radiculopathy, n (%)	1180	87	1267
No	700 (59.32)	43 (49.43)	743 (58.64)
Yes	480 (40.68)	44 (50.57)	524 (41.36)
Surgical Indication // Neurological: Myelopathy, n (%)	1180	87	1267
No	884 (74.92)	65 (74.71)	949 (74.90)
Yes	296 (25.08)	22 (25.29)	318 (25.10)
Surgical Indication // Neurological: High grade ESCC, n (%)	1180	87	1267
No	711 (60.25)	53 (60.92)	764 (60.30)
Yes	469 (39.75)	34 (39.08)	503 (39.70)
Surgical Indication // Oncologic: RT resistant, n (%)	1180	87	1267
No	1008 (85.42)	79 (90.80)	1087 (85.79)
Yes	172 (14.58)	8 (9.20)	180 (14.21)
Surgical Indication // Oncologic: Best known treatment, n (%)	1180	87	1267
No	412 (34.92)	40 (45.98)	452 (35.67)
Yes	768 (65.08)	47 (54.02)	815 (64.33)
Surgical Indication // Stability, n (%)	1087	79	1166
Stable	217 (19.96)	22 (27.85)	239 (20.50)
Impending instability	416 (38.27)	34 (43.04)	450 (38.59)
Unstable	454 (41.77)	23 (29.11)	477 (40.91)
Procedure type, n (%)	1158	87	1245
Palliative procedure	807 (69.69)	51 (58.62)	858 (68.92)
Curettage	271 (23.40)	28 (32.18)	299 (24.02)
En bloc	80 (6.91)	8 (9.20)	88 (7.07)

Table VIII. Summary of surgery variables by occurrence of postoperative AE

	Occurrence of any postoperative AE		
**Baseline characteristic**	**No N = 1022**	**Yes N = 245**	**Total N = 1267**
1.9 What was the surgical approach within this first surgical stage?, n (%)	1020	245	1265
Anterior	39 (3.82)	6 (2.45)	45 (3.56)
Posterior	971 (95.20)	228 (93.06)	1199 (94.78)
Both (simultaneous)	10 (0.98)	11 (4.49)	21 (1.66)
Number of stages, n (%)	1021	245	1266
1	1002 (98.14)	234 (95.51)	1236 (97.63)
2/3	19 (1.86)	11 (4.49)	30 (2.37)
1.5.1 Estimated blood loss during the procedure [ml]			
n	943	221	1164
Mean (sd)	619.25 (649.33)	857.52 (877.18)	664.49 (704.13)
Median (Q1; Q3)	500.00 (200.00; 800.00)	550.00 (400.00; 1000.00)	500.00 (250.00; 800.00)
Min; Max	0.00; 6000.00	0.00; 7500.00	0.00; 7500.00
Duration of first surgery (hours)			
n	1014	240	1254
Mean (sd)	3.24 (1.71)	4.23 (2.24)	3.43 (1.86)
Median (Q1; Q3)	2.92 (2.12; 3.92)	3.63 (2.68; 5.49)	3.05 (2.20; 4.08)
Min; Max	0.17; 11.93	0.32; 13.13	0.17; 13.13
Number of instrumented levels			
n	1022	245	1267
Mean (sd)	4.69 (2.69)	5.25 (3.08)	4.80 (2.78)
Median (Q1; Q3)	5.00 (3.00; 6.00)	5.00 (3.00; 7.00)	5.00 (3.00; 6.00)
Min; Max	0.00; 20.00	0.00; 17.00	0.00; 20.00
Surgical Indication // Neurological: Functional radiculopathy, n (%)	1022	245	1267
No	599 (58.61)	144 (58.78)	743 (58.64)
Yes	423 (41.39)	101 (41.22)	524 (41.36)
Surgical Indication // Neurological: Myelopathy, n (%)	1022	245	1267
No	780 (76.32)	169 (68.98)	949 (74.90)
Yes	242 (23.68)	76 (31.02)	318 (25.10)
Surgical Indication // Neurological: High grade ESCC, n (%)	1022	245	1267
No	622 (60.86)	142 (57.96)	764 (60.30)
Yes	400 (39.14)	103 (42.04)	503 (39.70)
Surgical Indication // Oncologic: RT resistant, n (%)	1022	245	1267
No	873 (85.42)	214 (87.35)	1087 (85.79)
Yes	149 (14.58)	31 (12.65)	180 (14.21)
Surgical Indication // Oncologic: Best known treatment, n (%)	1022	245	1267
No	361 (35.32)	91 (37.14)	452 (35.67)
Yes	661 (64.68)	154 (62.86)	815 (64.33)
Surgical Indication // Stability, n (%)	944	222	1166
Stable	189 (20.02)	50 (22.52)	239 (20.50)
Impending instability	366 (38.77)	84 (37.84)	450 (38.59)
Unstable	389 (41.21)	88 (39.64)	477 (40.91)
Procedure type, n (%)	1007	238	1245
Palliative procedure	717 (71.20)	141 (59.24)	858 (68.92)
Curettage	230 (22.84)	69 (28.99)	299 (24.02)
En bloc	60 (5.96)	28 (11.76)	88 (7.07)

.

Table IX. Summary of previous treatments by occurrence of intraoperative AE

	Occurrence of any intraoperative AE		
**Baseline characteristic**	**No<br>N = 1180**	**Yes<br>N = 87**	**Total<br>N = 1267**
Did the patient receive surgery to treat the index target PRIOR to inclusion?, n (%)	1180	87	1267
No	1081 (91.61)	72 (82.76)	1153 (91.00)
Yes	99 (8.39)	15 (17.24)	114 (9.00)
Did the patient receive radiation therapy to treat the index target PRIOR to inclusion?, n (%)	1179	87	1266
No	901 (76.42)	61 (70.11)	962 (75.99)
Yes	278 (23.58)	26 (29.89)	304 (24.01)
Did the patient receive systemic therapy to treat the metastatic disease PRIOR to inclusion?, n (%)	1179	87	1266
No	879 (74.55)	67 (77.01)	946 (74.72)
Yes	300 (25.45)	20 (22.99)	320 (25.28)

Table X. Summary of previous treatments by occurrence of postoperative AE

	Occurrence of any postoperative AE		
**Baseline characteristic**	**No<br>N = 1022**	**Yes<br>N = 245**	**Total<br>N = 1267**
Did the patient receive surgery to treat the index target PRIOR to inclusion?, n (%)	1022	245	1267
No	938 (91.78)	215 (87.76)	1153 (91.00)
Yes	84 (8.22)	30 (12.24)	114 (9.00)
Did the patient receive radiation therapy to treat the index target PRIOR to inclusion?, n (%)	1021	245	1266
No	800 (78.35)	162 (66.12)	962 (75.99)
Yes	221 (21.65)	83 (33.88)	304 (24.01)
Did the patient receive systemic therapy to treat the metastatic disease PRIOR to inclusion?, n (%)	1021	245	1266
No	771 (75.51)	175 (71.43)	946 (74.72)
Yes	250 (24.49)	70 (28.57)	320 (25.28)

Table XI. Summary of logistic regression model for occurrence of any intraoperative AE

						Wald 95% confidence limits for estimate				Wald 95% confidence limits for OR	
**Category of variable**	**Variable**	**Reference**	**Level**	**Estimate**	**Standard Error**	**Lower**	**Upper**	**P-value**	**Odds Ratio**	**Lower**	**Upper**
	Intercept			-4.692	0.907	-6.469	-2.914	<.0001	.	.	.
Demographics	Age at baseline (years)			0.016	0.011	-0.006	0.038	0.1620	1.016	0.994	1.038
	Gender	Male	Female	-0.023	0.130	-0.278	0.232	0.8602	0.955	0.574	1.591
Neurological status	ECOG Performance Status	0	1/2	0.156	0.182	-0.200	0.513	0.3894	1.252	0.591	2.653
			3/4	-0.088	0.231	-0.541	0.364	0.7028	0.981	0.402	2.394
	Total SINS score	Stable: 0-6	Indeterminate: 7-12	-0.322	0.175	-0.665	0.021	0.0655	0.400	0.202	0.790
			Unstable: 13-18	-0.272	0.217	-0.697	0.152	0.2087	0.420	0.187	0.942
Surgery	Duration of first surgery (hours)			0.136	0.070	-0.001	0.274	0.0510	1.146	0.999	1.315
	Estimated blood loss			0.001	0.000	0.001	0.001	<.0001	1.001	1.001	1.001
	Procedure type	Palliative procedure	Curettage	0.190	0.219	-0.239	0.619	0.3854	1.092	0.620	1.923
			En bloc	-0.292	0.324	-0.927	0.344	0.3681	0.674	0.250	1.816
	Surgical approach	Anterior	Both (simultaneous)	-0.512	0.585	-1.658	0.634	0.3809	0.338	0.048	2.382
			Posterior	-0.060	0.345	-0.736	0.615	0.8608	0.531	0.174	1.618
	Surgical indication // High grade ESCC	No	Yes	-0.055	0.132	-0.313	0.204	0.6781	0.896	0.535	1.502

N=1045 (76 subjects with intraoperative AE, 969 subjects without intraoperative AE) observations used for model estimation.

Table XII. Summary of logistic regression model for occurrence of any postoperative AE

						Wald 95% confidence limits for estimate				Wald 95% confidence limits for OR	
**Category of variable**	**Variable**	**Reference**	**Level**	**Estimate**	**Standard Error**	**Lower**	**Upper**	**P-value**	**Odds Ratio**	**Lower**	**Upper**
	Intercept			-3.193	0.775	-4.713	-1.673	<.0001	.	.	.
Demographics	Age at baseline (years)			0.022	0.008	0.007	0.037	0.0044	1.022	1.007	1.038
	Charlson Comorbidity Index			-0.011	0.065	-0.138	0.115	0.8629	0.989	0.871	1.122
	Gender	Male	Female	0.023	0.088	-0.149	0.196	0.7901	1.048	0.742	1.481
	Smoking status	No	Not assessed	-0.386	0.178	-0.735	-0.037	0.0302	0.858	0.536	1.371
			Yes - currently	0.501	0.214	0.083	0.920	0.0189	2.083	1.170	3.709
			Yes - previously	0.117	0.158	-0.193	0.427	0.4590	1.418	0.931	2.162
Neurological status	ECOG Performance Status	0	1/2	0.102	0.131	-0.155	0.360	0.4353	1.823	1.001	3.319
			3/4	0.396	0.155	0.093	0.699	0.0105	2.445	1.263	4.733
	Total SINS score	Stable: 0-6	Indeterminate: 7-12	-0.012	0.132	-0.270	0.246	0.9257	1.206	0.661	2.200
			Unstable: 13-18	0.211	0.158	-0.098	0.521	0.1810	1.508	0.771	2.950
Previous treatment	Did the patient receive radiation therapy to treat the index target PRIOR to inclusion?	No	Yes	0.372	0.106	0.165	0.580	0.0004	2.105	1.391	3.188
	Did the patient receive surgery to treat the index target PRIOR to inclusion?	No	Yes	0.126	0.148	-0.163	0.415	0.3936	1.286	0.721	2.294
	Did the patient receive systemic therapy to treat the metastatic disease PRIOR to inclusion?	No	Yes	-0.124	0.116	-0.352	0.104	0.2854	0.780	0.495	1.230
Surgery	Duration of first surgery (hours)			0.196	0.054	0.090	0.302	0.0003	1.216	1.094	1.352
	Estimated blood loss			0.000	0.000	0.000	0.001	0.0057	1.000	1.000	1.001
	Number of instrumented levels			-0.035	0.037	-0.107	0.036	0.3338	0.965	0.899	1.037
	Number of stages	1	2/3	0.637	0.281	0.087	1.188	0.0233	3.576	1.189	10.756
	Procedure type	Palliative procedure	Curettage	-0.076	0.147	-0.363	0.211	0.6033	1.192	0.801	1.774
			En bloc	0.328	0.212	-0.087	0.743	0.1218	1.784	0.928	3.432
	Surgical approach	Anterior	Both (simultaneous)	0.935	0.410	0.131	1.738	0.0227	7.535	1.484	38.261
			Posterior	0.151	0.295	-0.427	0.728	0.6097	3.440	0.933	12.690
	Surgical indication // High grade ESCC	No	Yes	-0.055	0.088	-0.228	0.117	0.5301	0.895	0.634	1.265
Tumor information	Does the patient have metastases at other site(s)?	No	Yes	0.233	0.093	0.051	0.416	0.0120	1.595	1.108	2.296
	Location of metastatic spine tumor in C	No	Yes	-0.221	0.133	-0.482	0.040	0.0963	0.643	0.381	1.082
	Location of metastatic spine tumor in L	No	Yes	-0.259	0.114	-0.482	-0.036	0.0230	0.596	0.382	0.931

	Location of metastatic spine tumor in S	No	Yes	-0.025	0.162	-0.343	0.294	0.8795	0.952	0.504	1.799
	Location of metastatic spine tumor in T	No	Yes	-0.112	0.128	-0.363	0.140	0.3833	0.800	0.484	1.322
	Number of spine metastases			0.076	0.031	0.015	0.137	0.0141	1.079	1.015	1.147

N=1044 (200 subjects with postoperative AE, 844 subjects without postoperative AE) observations used for model estimation.

Table XIII. Summary of logistic regression model for occurrence of surgery-related AE

						Wald 95% confidence limits for estimate				Wald 95% confidence limits for OR	
**Category of variable**	**Variable**	**Reference**	**Level**	**Estimate**	**Standard Error**	**Lower**	**Upper**	**P-value**	**Odds Ratio**	**Lower**	**Upper**
	Intercept			-2.170	0.728	-3.598	-0.743	0.0029	.	.	.
Demographics	Age at baseline (years)			0.023	0.007	0.009	0.037	0.0015	1.023	1.009	1.038
	Charlson Comorbidity Index			-0.025	0.062	-0.146	0.096	0.6901	0.976	0.865	1.101
	Gender	Male	Female	0.047	0.082	-0.114	0.209	0.5673	1.099	0.796	1.518
	Smoking status	No	Not assessed	-0.199	0.163	-0.518	0.120	0.2214	0.983	0.644	1.499
			Yes - currently	0.386	0.209	-0.025	0.796	0.0654	1.764	1.004	3.100
			Yes - previously	-0.005	0.154	-0.307	0.298	0.9752	1.194	0.795	1.793
Neurological status	ASIA Impairment Scale	D/E	A/B/C	0.039	0.140	-0.236	0.313	0.7816	1.081	0.624	1.871
	ECOG Performance Status	0	1/2	0.092	0.121	-0.146	0.330	0.4470	1.648	0.965	2.815
			3/4	0.315	0.154	0.014	0.616	0.0404	2.058	1.103	3.838
	Total SINS score	Stable: 0-6	Indeterminate: 7-12	-0.105	0.120	-0.340	0.130	0.3827	0.918	0.539	1.562
			Unstable: 13-18	0.123	0.146	-0.162	0.409	0.3980	1.152	0.631	2.104
Previous treatment	Did the patient receive radiation therapy to treat the index target PRIOR to inclusion?	No	Yes	0.318	0.101	0.119	0.516	0.0017	1.888	1.269	2.809
	Did the patient receive surgery to treat the index target PRIOR to inclusion?	No	Yes	0.150	0.140	-0.124	0.424	0.2824	1.350	0.781	2.333
	Did the patient receive systemic therapy to treat the metastatic disease PRIOR to inclusion?	No	Yes	-0.119	0.110	-0.335	0.096	0.2771	0.787	0.512	1.212
Surgery	Duration of first surgery (hours)			0.205	0.052	0.102	0.307	<.0001	1.227	1.108	1.359
	Estimated blood loss			0.000	0.000	0.000	0.001	<.0001	1.000	1.000	1.001
	Number of instrumented levels			-0.045	0.034	-0.112	0.023	0.1925	0.956	0.894	1.023
	Number of stages	1	2/3	1.060	0.267	0.536	1.583	<.0001	8.325	2.920	23.732
	Procedure type	Palliative procedure	Curettage	0.006	0.141	-0.270	0.281	0.9687	1.179	0.814	1.710
			En bloc	0.154	0.207	-0.252	0.560	0.4571	1.368	0.724	2.585
	Surgical approach	Anterior	Both (simultaneous)	0.806	0.399	0.025	1.587	0.0432	5.095	1.148	22.622
			Posterior	0.017	0.270	-0.513	0.546	0.9508	2.314	0.763	7.024
	Surgical indication // High grade ESCC	No	Yes	-0.031	0.082	-0.192	0.130	0.7076	0.940	0.681	1.298
Tumor information	Does the patient have metastases at other site(s)?	No	Yes	0.038	0.112	-0.182	0.258	0.7331	1.079	0.696	1.675
	Location of metastatic spine tumor in C	No	Yes	-0.220	0.126	-0.466	0.027	0.0809	0.644	0.393	1.056

	Location of metastatic spine tumor in L	No	Yes	-0.213	0.105	-0.419	-0.006	0.0436	0.654	0.433	0.988
	Location of metastatic spine tumor in S	No	Yes	0.143	0.147	-0.146	0.431	0.3319	1.331	0.747	2.369
	Location of metastatic spine tumor in T	No	Yes	-0.104	0.119	-0.337	0.128	0.3788	0.812	0.510	1.292
	Number of spine metastases			0.065	0.029	0.007	0.123	0.0274	1.067	1.007	1.130
	Visceral/brain metastases	No	Yes	0.190	0.111	-0.027	0.407	0.0861	1.463	0.947	2.258

N=1044 (247 subjects with surgery-related AE, 797 subjects without surgery-related AE) observations used

Table XIV. Summary of Cox-model for survival with occurrence of intraoperative AE as input variable

								95% Confidence Limit for Hazard Ratio	
**Vargroup**	**Variable**	**Reference**	**Level**	**Parameter Estimate**	**Standard Error**	**P-value**	**Hazard Ratio**	**Lower Limit**	**Upper Limit**
AE	Occurrence of any intraoperative AE	No	Yes	-0.115	0.171	0.5036	0.892	0.637	1.248
Demographics	Age at baseline			0.006	0.004	0.1187	1.006	0.998	1.014
	Charlson Comorbidity Index (Version 2)			0.102	0.034	0.0024	1.107	1.037	1.183
	Gender	Male	Female	-0.142	0.096	0.1397	0.868	0.719	1.047
	Smoking status	No	Not assessed	0.196	0.125	0.1162	1.216	0.953	1.553
			Yes - currently	0.013	0.172	0.9381	1.013	0.723	1.420
			Yes - previously	0.379	0.113	0.0008	1.460	1.170	1.823
Neurological status	ASIA impairment scale	D/E	A/B/C	0.259	0.157	0.0996	1.296	0.952	1.763
	ECOG Performance Status	0	1/2	0.323	0.164	0.0497	1.381	1.000	1.905
			3/4	0.422	0.188	0.0250	1.526	1.055	2.207
	Total SINS score	Stable: 0-6	Indeterminate: 7-12	0.217	0.181	0.2307	1.243	0.871	1.773
			Unstable: 13-18	0.113	0.204	0.5816	1.119	0.750	1.670
Prior treatment	Did the patient receive radiation therapy to treat the index target PRIOR to inclusion?	No	Yes	-0.123	0.121	0.3078	0.884	0.698	1.120
	Did the patient receive surgery to treat the index target PRIOR to inclusion?	No	Yes	-0.359	0.189	0.0575	0.698	0.482	1.012
	Did the patient receive systemic therapy to treat the metastatic disease PRIOR to inclusion?	No	Yes	0.196	0.122	0.1062	1.217	0.959	1.545
Surgery	Duration of first surgery			0.001	0.035	0.9841	1.001	0.935	1.071
	Estimated blood loss during the procedure (mL)			-0.000	0.000	0.7757	1.000	1.000	1.000
	Number of instrumented levels			0.012	0.022	0.5791	1.012	0.970	1.057
	Number of stages	1	2/3	0.113	0.351	0.7482	1.119	0.563	2.225
	Procedure type	Palliative	Curettage	-0.467	0.123	0.0002	0.627	0.492	0.798
			En bloc	-0.459	0.234	0.0496	0.632	0.399	0.999
	Surgical Indication // Neurological: Functional radiculopathy	No	Yes	0.162	0.098	0.0992	1.176	0.970	1.426
	Surgical Indication // Neurological: High grade ESCC	No	Yes	0.291	0.093	0.0017	1.338	1.115	1.605
	Surgical Indication // Neurological: Myelopathy	No	Yes	0.226	0.106	0.0340	1.253	1.017	1.544
	Surgical Indication // Oncologic: Best known treatment	No	Yes	-0.193	0.102	0.0587	0.824	0.675	1.007
	Surgical Indication // Oncologic: RT resistant	No	Yes	-0.026	0.137	0.8512	0.975	0.745	1.274
	Surgical Indication // Stability	Stable	Impending instability	0.070	0.137	0.6102	1.072	0.820	1.403

								95% Confidence Limit for Hazard Ratio	
**Vargroup**	**Variable**	**Reference**	**Level**	**Parameter Estimate**	**Standard Error**	**P-value**	**Hazard Ratio**	**Lower Limit**	**Upper Limit**
			Unstable	0.335	0.142	0.0188	1.398	1.057	1.848
	Surgical approach within the first surgical approach	Posterior	Anterior	-0.153	0.320	0.6334	0.858	0.458	1.608
			Both (simultaneous)	-0.705	0.446	0.1138	0.494	0.206	1.184
Tumor information	Does the patient have metastases at other site(s)?	No	Yes	0.144	0.128	0.2578	1.155	0.900	1.484
	Location of metastatic spine tumor in C	No	Yes	-0.025	0.132	0.8493	0.975	0.752	1.264
	Location of metastatic spine tumor in L	No	Yes	-0.067	0.117	0.5674	0.935	0.743	1.177
	Location of metastatic spine tumor in S	No	Yes	0.236	0.164	0.1495	1.266	0.919	1.745
	Location of metastatic spine tumor in T	No	Yes	0.160	0.133	0.2308	1.173	0.903	1.524
	Number of spine metastases			-0.005	0.016	0.7429	0.995	0.964	1.027
	Visceral/brain metastases	No	Yes	0.526	0.122	<.0001	1.693	1.333	2.150

Note: N = 985 (Event = 530, Censored = 455) observations used for model estimation.

Table XV. Summary of Cox-model for survival including postoperative AE as input variable

								95% Confidence Limit for Hazard Ratio	
Vargroup	Variable	Reference	Level	Parameter Estimate	Standard Error	P-value	Hazard Ratio	Lower Limit	Upper Limit
AE	Occurrence of any postoperative AE	No	Yes	0.175	0.116	0.1289	1.192	0.950	1.494
Demographics	Age at baseline			0.005	0.004	0.1862	1.005	0.997	1.013
	Charlson Comorbidity Index (Version 2)			0.100	0.034	0.0031	1.105	1.034	1.180
	Gender	Male	Female	-0.140	0.096	0.1454	0.870	0.721	1.050
	Smoking status	No	Not assessed	0.199	0.125	0.1096	1.221	0.956	1.558
			Yes - currently	-0.009	0.173	0.9571	0.991	0.706	1.391
			Yes - previously	0.373	0.113	0.0010	1.452	1.162	1.813
Neurological status	ASIA impairment scale	D/E	A/B/C	0.246	0.158	0.1189	1.279	0.939	1.744
	ECOG Performance Status	0	1/2	0.313	0.164	0.0567	1.367	0.991	1.887
			3/4	0.411	0.189	0.0293	1.509	1.042	2.184
	Total SINS score	Stable: 0-6	Indeterminate: 7-12	0.238	0.181	0.1886	1.268	0.890	1.808
			Unstable: 13-18	0.124	0.204	0.5451	1.132	0.758	1.688
Prior treatment	Did the patient receive radiation therapy to treat the index target PRIOR to inclusion?	No	Yes	-0.143	0.122	0.2404	0.867	0.683	1.100
	Did the patient receive surgery to treat the index target PRIOR to inclusion?	No	Yes	-0.349	0.189	0.0651	0.705	0.487	1.022
	Did the patient receive systemic therapy to treat the metastatic disease PRIOR to inclusion?	No	Yes	0.206	0.122	0.0910	1.228	0.968	1.559
Surgery	Duration of first surgery			-0.006	0.035	0.8637	0.994	0.928	1.065
	Estimated blood loss during the procedure (mL)			-0.000	0.000	0.6132	1.000	1.000	1.000
	Number of instrumented levels			0.012	0.022	0.5894	1.012	0.969	1.056
	Number of stages	1	2/3	0.049	0.348	0.8872	1.051	0.531	2.079
	Procedure type	Palliative	Curettage	-0.469	0.123	0.0001	0.625	0.491	0.797
			En bloc	-0.467	0.234	0.0464	0.627	0.396	0.993
	Surgical Indication // Neurological: Functional radiculopathy	No	Yes	0.157	0.098	0.1082	1.170	0.966	1.416
	Surgical Indication // Neurological: High grade ESCC	No	Yes	0.295	0.093	0.0015	1.342	1.119	1.611
	Surgical Indication // Neurological: Myelopathy	No	Yes	0.221	0.107	0.0387	1.247	1.012	1.537
	Surgical Indication // Oncologic: Best known treatment	No	Yes	-0.181	0.101	0.0749	0.835	0.684	1.018
	Surgical Indication // Oncologic: RT resistant	No	Yes	-0.014	0.137	0.9199	0.986	0.755	1.289

								95% Confidence Limit for Hazard Ratio	
**Vargroup**	**Variable**	**Reference**	**Level**	**Parameter Estimate**	**Standard Error**	**P-value**	**Hazard Ratio**	**Lower Limit**	**Upper Limit**
	Surgical Indication // Stability	Stable	Impending instability	0.079	0.137	0.5618	1.083	0.828	1.416
			Unstable	0.339	0.142	0.0172	1.404	1.062	1.857
	Surgical approach within the first surgical approach	Posterior	Anterior	-0.121	0.319	0.7044	0.886	0.474	1.656
			Both (simultaneous)	-0.763	0.448	0.0885	0.466	0.194	1.122
Tumor information	Does the patient have metastases at other site(s)?	No	Yes	0.127	0.128	0.3211	1.136	0.883	1.460
	Location of metastatic spine tumor in C	No	Yes	-0.021	0.132	0.8717	0.979	0.755	1.269
	Location of metastatic spine tumor in L	No	Yes	-0.079	0.117	0.5016	0.924	0.734	1.163
	Location of metastatic spine tumor in S	No	Yes	0.244	0.164	0.1367	1.276	0.926	1.758
	Location of metastatic spine tumor in T	No	Yes	0.153	0.133	0.2528	1.165	0.897	1.513
	Number of spine metastases			-0.007	0.016	0.6748	0.993	0.962	1.025
	Visceral/brain metastases	No	Yes	0.519	0.122	<.0001	1.680	1.322	2.133

Note: N = 985 (Event = 530, Censored = 455) observations used for model estimation

Table XVI. Summary of Cox-model for survival with occurrence of any surgery-related AE (intraop or postop) as input variable

								95% Confidence Limit for Hazard Ratio	
**Vargroup**	**Variable**	**Reference**	**Level**	**Parameter Estimate**	**Standard Error**	**P-value**	**Hazard Ratio**	**Lower Limit**	**Upper Limit**
AE	Occurrence of any surgery-related AE (intraop or postop)	No	Yes	0.059	0.108	0.5817	1.061	0.859	1.311
Demographics	Age at baseline			0.006	0.004	0.1396	1.006	0.998	1.014
	Charlson Comorbidity Index (Version 2)			0.102	0.034	0.0024	1.108	1.037	1.183
	Gender	Male	Female	-0.139	0.096	0.1484	0.871	0.721	1.051
	Smoking status	No	Not assessed	0.192	0.124	0.1235	1.211	0.949	1.546
			Yes - currently	0.012	0.172	0.9444	1.012	0.722	1.419
			Yes - previously	0.377	0.113	0.0009	1.458	1.167	1.820
Neurological status	ASIA impairment scale	D/E	A/B/C	0.253	0.158	0.1087	1.289	0.945	1.756
	ECOG Performance Status	0	1/2	0.314	0.164	0.0561	1.369	0.992	1.889
			3/4	0.414	0.189	0.0282	1.513	1.045	2.190
	Total SINS score	Stable: 0-6	Indeterminate: 7-12	0.235	0.181	0.1946	1.265	0.887	1.803
			Unstable: 13-18	0.125	0.204	0.5407	1.133	0.760	1.689
Prior treatment	Did the patient receive radiation therapy to treat the index target PRIOR to inclusion?	No	Yes	-0.129	0.121	0.2854	0.879	0.693	1.114
	Did the patient receive surgery to treat the index target PRIOR to inclusion?	No	Yes	-0.355	0.189	0.0611	0.701	0.484	1.017
	Did the patient receive systemic therapy to treat the metastatic disease PRIOR to inclusion?	No	Yes	0.203	0.122	0.0950	1.225	0.965	1.555
Surgery	Duration of first surgery			-0.001	0.035	0.9697	0.999	0.932	1.070
	Estimated blood loss during the procedure (mL)			-0.000	0.000	0.6276	1.000	1.000	1.000
	Number of instrumented levels			0.012	0.022	0.5849	1.012	0.970	1.056
	Number of stages	1	2/3	0.065	0.351	0.8533	1.067	0.536	2.124
	Procedure type	Palliative	Curettage	-0.471	0.123	0.0001	0.624	0.490	0.795
			En bloc	-0.457	0.234	0.0511	0.633	0.400	1.002
	Surgical Indication // Neurological: Functional radiculopathy	No	Yes	0.151	0.098	0.1210	1.163	0.961	1.409
	Surgical Indication // Neurological: High grade ESCC	No	Yes	0.290	0.093	0.0018	1.337	1.114	1.604
	Surgical Indication // Neurological: Myelopathy	No	Yes	0.226	0.107	0.0344	1.253	1.017	1.544
	Surgical Indication // Oncologic: Best known treatment	No	Yes	-0.181	0.102	0.0747	0.834	0.684	1.018
	Surgical Indication // Oncologic: RT resistant	No	Yes	-0.013	0.137	0.9267	0.988	0.756	1.290

								95% Confidence Limit for Hazard Ratio	
**Vargroup**	**Variable**	**Reference**	**Level**	**Parameter Estimate**	**Standard Error**	**P-value**	**Hazard Ratio**	**Lower Limit**	**Upper Limit**
	Surgical Indication // Stability	Stable	Impending instability	0.075	0.137	0.5829	1.078	0.824	1.410
			Unstable	0.341	0.142	0.0167	1.406	1.064	1.859
	Surgical approach within the first surgical approach	Posterior	Anterior	-0.136	0.320	0.6716	0.873	0.466	1.635
			Both (simultaneous)	-0.717	0.448	0.1096	0.488	0.203	1.175
Tumor information	Does the patient have metastases at other site(s)?	No	Yes	0.136	0.128	0.2879	1.146	0.891	1.473
	Location of metastatic spine tumor in C	No	Yes	-0.028	0.132	0.8324	0.972	0.751	1.260
	Location of metastatic spine tumor in L	No	Yes	-0.076	0.118	0.5205	0.927	0.736	1.167
	Location of metastatic spine tumor in S	No	Yes	0.238	0.164	0.1461	1.269	0.920	1.749
	Location of metastatic spine tumor in T	No	Yes	0.153	0.133	0.2515	1.165	0.897	1.514
	Number of spine metastases			-0.006	0.016	0.7315	0.994	0.964	1.026
	Visceral/brain metastases	No	Yes	0.520	0.122	<.0001	1.682	1.324	2.137

Note: N = 985 (Event = 530, Censored = 455) observations used for model estimation.

Table XVII. Summary of SOSGOQ mean values (for each domain) including standard deviations and p-values by occurrence of AEs

	**Occurrence of any intraoperative AE**		
**Characteristic**	**No**<br>**N = 1180**	**Yes**<br>**N = 87**	***P* value**
Total SOSGOQ score at baseline			
Mean (sd)	53.72 (20.71)	48.03 (19.74)	0.1528
Total SOSGOQ score at two months			
Mean (sd)	65.24 (16.91)	67.96 (17.18)	0.5298
Total SOSGOQ score at six months			
Mean (sd)	72.10 (18.02)	71.69 (18.56)	0.9389
P values between timepoints			
Baseline vs. 2-month FU	**<.0001**	**0.0012**	
Baseline vs. 6-month FU	**<.0001**	**0.0007**	
2-month FU vs. 6-month	**0.0009**[¶]	0.5731	
Physical function domain score at baseline			
Mean (sd)	53.23 (27.80)	47.52 (24.70)	0.2824
Physical function domain score at two months			
Mean (sd)	55.46 (22.57)	60.76 (18.14)	0.3492
Physical function domain score at six months			
Mean (sd)	68.03 (22.60)	63.15 (17.78)	0.4521
P values between timepoints			
Baseline vs. 2-month FU	0.3565	0.0608	
Baseline vs. 6-month FU	**<.0001**	**0.0469**	
2-month FU vs. 6-month	**<.0001**	0.7212	
Neurological function domain score at baseline			
Mean (sd)	80.65 (21.00)	78.48 (20.37)	0.5918
Neurological function domain score at two months			
Mean (sd)	83.94 (17.07)	87.94 (13.90)	0.3504
Neurological function domain score at six months			
Mean (sd)	85.27 (18.53)	90.92 (10.73)	0.1117
P values between timepoints			
Baseline vs. 2-month FU	0.0714	0.0975	
Baseline vs. 6-month FU	**0.0263**	**0.0452**	
2-month FU vs. 6-month	0.5187	0.5272	
Pain domain score at baseline			
Mean (sd)	38.73 (25.40)	36.69 (23.98)	0.6760
Pain domain score at two months			
Mean (sd)	60.56 (20.89)	65.65 (21.83)	0.3414
Pain domain score at six months			
Mean (sd)	67.80 (21.64)	69.85 (26.02)	0.7509
P values between timepoints			
Baseline vs. 2-month FU	**<.0001**	**0.0002**	
Baseline vs. 6-month FU	**<.0001**	**0.0002**	
2-month FU vs. 6-month	**0.0038**	0.6346	
Mental domain score at baseline			
Mean (sd)	60.44 (27.64)	50.21 (27.89)	0.0552
Mental domain score at two months			
Mean (sd)	73.02 (25.03)	65.53 (25.96)	0.2422
Mental domain score at six months			
Mean (sd)	74.90 (23.78)	78.00 (30.19)	0.6629
P values between timepoints			
Baseline vs. 2-month FU	**<.0001**	0.0719	
Baseline vs. 6-month FU	**<.0001**	**0.0059**	
2-month FU vs. 6-month	0.5120	0.2344	
Social domain score at baseline			
Mean (sd)	62.05 (25.22)	57.69 (22.47)	0.3666

	Occurrence of any intraoperative AE		
**Characteristic**	**No<br>N = 1180**	**Yes<br>N = 87**	***P* value**
Social domain score at two months			
Mean (sd)	71.15 (20.57)	79.88 (21.53)	0.0982
Social domain score at six months			
Mean (sd)	77.38 (20.37)	75.77 (18.07)	0.7850
P values between timepoints			
Baseline vs. 2-month FU	**<.0001**	**0.0020**	
Baseline vs. 6-month FU	**<.0001**	**0.0147**	
2-month FU vs. 6-month	**0.0098**	0.5834	
	**Occurrence of any postoperative AE**		
**Characteristic**	**No<br>N = 1022**	**Yes<br>N = 245**	***P* value**
Total SOSGOQ score at baseline			
Mean (sd)	54.28 (20.92)	50.89 (19.94)	0.129
Total SOSGOQ score at two months			
Mean (sd)	67.40 (16.78)	60.09 (16.23)	**0.0097**
Total SOSGOQ score at six months			
Mean (sd)	74.66 (16.25)	66.73 (20.29)	**0.0138**
P values between timepoints			
Baseline vs. 2-month FU	**<.0001**	**0.0051**	
Baseline vs. 6-month FU	**<.0001**	**<.0001**	
2-month FU vs. 6-month	**0.0012**	0.0825	
Physical function domain score at baseline			
Mean (sd)	55.27 (27.14)	46.70 (27.94)	**0.0037**
Physical function domain score at two months			
Mean (sd)	58.82 (21.50)	48.04 (22.45)	**0.0031**
Physical function domain score at six months			
Mean (sd)	71.47 (20.79)	59.72 (23.08)	**0.0027**
P values between timepoints			
Baseline vs. 2-month FU	0.1765	0.7641	
Baseline vs. 6-month FU	**<.0001**	**0.0051**	
2-month FU vs. 6-month	**<.0001**	**0.0131**	
Neurological function domain score at baseline			
Mean (sd)	82.11 (19.78)	76.45 (23.21)	**0.0193**
Neurological function domain score at two months			
Mean (sd)	84.67 (16.65)	83.26 (17.40)	0.6118
Neurological function domain score at six months			
Mean (sd)	87.25 (17.73)	82.78 (18.39)	0.1678
P values between timepoints			
Baseline vs. 2-month FU	0.1859	0.0637	
Baseline vs. 6-month FU	**0.0240**	0.0987	
2-month FU vs. 6-month	0.2591	0.8963	
Pain domain score at baseline			
Mean (sd)	39.73 (25.50)	35.69 (24.57)	0.1385
Pain domain score at two months			
Mean (sd)	61.88 (21.43)	58.66 (19.66)	0.3535
Pain domain score at six months			
Mean (sd)	70.47 (20.69)	63.04 (23.83)	0.0584
P values between timepoints			
Baseline vs. 2-month FU	**<.0001**	**<.0001**	
Baseline vs. 6-month FU	**<.0001**	**<.0001**	
2-month FU vs. 6-month	**0.0027**	0.3244	
Mental domain score at baseline			
Mean (sd)	59.65 (27.45)	59.97 (28.60)	0.9157
Mental domain score at two months			
Mean (sd)	74.79 (24.17)	65.41 (26.76)	**0.0243**

	Occurrence of any intraoperative AE		
**Characteristic**	**No**<br>**N = 1180**	**Yes**<br>**N = 87**	***P* value**
Mental domain score at six months			
Mean (sd)	78.23 (22.01)	69.02 (27.67)	**0.0333**
P values between timepoints			
Baseline vs. 2-month FU	**<.0001**	0.2553	
Baseline vs. 6-month FU	**<.0001**	0.0655	
2-month FU vs. 6-month	0.2689	0.5170[¶]	
Social domain score at baseline			
Mean (sd)	62.37 (25.07)	60.17 (25.01)	0.4147
Social domain score at two months			
Mean (sd)	73.26 (21.38)	68.20 (18.56)	0.1430
Social domain score at six months			
Mean (sd)	78.88 (19.76)	73.80 (20.61)	0.1604
P values between timepoints			
Baseline vs. 2-month FU	**<.0001**	**0.0438**	
Baseline vs. 6-month FU	**<.0001**	**0.0012**	
2-month FU vs. 6-month	**0.0427**	0.1668	
Post therapy domain score at two months			

	Occurrence of any surgery-related AE		
**Characteristic**	**No**<br>**N = 968**	**Yes**<br>**N = 299**	***P* value**
Total SOSGOQ score at baseline			
Mean (sd)	54.64 (20.90)	50.27 (19.91)	**0.0461**
Total SOSGOQ score at two months			
Mean (sd)	66.90 (16.83)	62.01 (16.73)	0.0753
Total SOSGOQ score at six months			
Mean (sd)	74.45 (16.20)	67.61 (20.38)	**0.0315**
P values between timepoints			
Baseline vs. 2-month FU	**<.0001**	**0.0002**	
Baseline vs. 6-month FU	**<.0001**	**<.0001**	
2-month FU vs. 6-month	**0.0010**	0.1311	
Physical function domain score at baseline			
Mean (sd)	55.52 (27.02)	46.65 (28.07)	**0.0022**
Physical function domain score at two months			
Mean (sd)	58.28 (21.51)	50.33 (23.07)	**0.0253**
Physical function domain score at six months			
Mean (sd)	71.50 (21.04)	60.38 (22.65)	**0.0040**
P values between timepoints			
Baseline vs. 2-month FU	0.3009	0.3934	
Baseline vs. 6-month FU	**<.0001**	**0.0023**	
2-month FU vs. 6-month	**<.0001**	**0.0266**	
Neurological function domain score at baseline			
Mean (sd)	82.15 (19.67)	76.71 (23.25)	**0.0211**
Neurological function domain score at two months			
Mean (sd)	84.38 (16.83)	84.11 (16.94)	0.9215
Neurological function domain score at six months			
Mean (sd)	86.97 (17.92)	83.59 (18.15)	0.2907
P values between timepoints			
Baseline vs. 2-month FU	0.2587	**0.0344**	
Baseline vs. 6-month FU	**0.0370**	0.0635	
2-month FU vs. 6-month	0.2700	0.8808	
Pain domain score at baseline			
Mean (sd)	40.01 (25.67)	35.30 (24.14)	0.0779
Pain domain score at two months			
Mean (sd)	61.27 (21.56)	60.44 (19.63)	0.8062
Pain domain score at six months			
Mean (sd)	70.07 (20.68)	64.22 (23.92)	0.1313
P values between timepoints			

	**Occurrence of any intraoperative AE**		
**Characteristic**	**No**<br>**N = 1180**	**Yes**<br>**N = 87**	***P* value**
Baseline vs. 2-month FU	**<.0001**	**<.0001**	
Baseline vs. 6-month FU	**<.0001**	**<.0001**	
2-month FU vs. 6-month	**0.0026**	0.3760¶	
Mental domain score at baseline			
Mean (sd)	60.07 (27.46)	58.96 (28.51)	0.7065
Mental domain score at two months			
Mean (sd)	74.78 (24.29)	66.31 (26.41)	**0.0364**
Mental domain score at six months			
Mean (sd)	77.92 (22.08)	70.14 (27.52)	0.0684
P values between timepoints			
Baseline vs. 2-month FU	**<.0001**	**0.1066**	
Baseline vs. 6-month FU	**<.0001**	**0.0188**	
2-month FU vs. 6-month	0.3218	0.4712	
Social domain score at baseline			
Mean (sd)	62.85 (25.08)	59.20 (24.86)	0.1691
Social domain score at two months			
Mean (sd)	72.45 (21.31)	70.69 (19.44)	0.5996
Social domain score at six months			
Mean (sd)	78.74 (19.61)	74.39 (20.93)	0.2224
P values between timepoints			
Baseline vs. 2-month FU	**0.0001**	**0.0029**	
Baseline vs. 6-month FU	**<.0001**	**0.0002**	
2-month FU vs. 6-month	**0.0253**	0.3542	

P values are calculated by t test

Table XVIII. Summary of MMRM model for difference of SOSGOQ physical function domain score and AE as input variable

						**95% Confidence Limit**		
**Intraoperative AEs as input variable**								
**Variable Group**	**Variable**	**Reference**	**Level**	**Estimate**	**Standard Error**	**Lower Limit**	**Upper Limit**	**P-value**
	Intercept			55.170	16.273	23.039	87.301	0.001
Neurological status	ASIA Impairment Scale	D/E	A/B/C	-12.967	6.742	-26.285	0.351	**0.056**
SOSGOQ	Baseline physical function domain score			-0.621	0.070	-0.759	-0.484	0.000
Surgery	Procedure type	Palliative procedure	Curettage	9.209	3.532	2.231	16.187	**0.010**
Tumor information	Visceral/brain metastases	No	Yes	9.174	4.526	0.231	18.117	**0.044**
time	Timepoint	2 Months	6 Months	7.402	1.995	3.444	11.360	0.000
**Postoperative AEs as input variable**								
**Variable Group**	**Variable**	**Reference**	**Level**	**Estimate**	**Standard Error**	**Lower Limit**	**Upper Limit**	**P-value**
	Intercept			56.898	16.087	25.132	88.663	0.001
AE	Occurrence of any postoperative AE	No	Yes	-6.649	3.447	-13.462	0.164	**0.056**
Neurological status	ASIA Impairment Scale	D/E	A/B/C	-14.526	6.604	-27.574	-1.478	**0.029**
SOSGOQ	Baseline physical function domain score			-0.664	0.069	-0.801	-0.527	0.000
Surgery	Procedure type	Palliative procedure	Curettage	9.135	3.488	2.243	16.028	**0.010**
Tumor information	Does the patient have metastases at other site(s)?	No	Yes	-9.117	4.229	-17.473	-0.761	**0.033**
	Visceral/brain metastases	No	Yes	9.320	4.456	0.514	18.126	**0.038**
time	Timepoint	2 Months	6 Months	7.503	2.005	3.524	11.482	0.000

Table XIX. Summary of MMRM model for difference of SOSGOQ neurological domain score and AE as input variable

						**95% Confidence Limit**		
**Intraoperative AEs as input variable**								
**Variable Group**	**Variable**	**Reference**	**Level**	**Estimate**	**Standard Error**	**Lower Limit**	**Upper Limit**	**P-value**
	Intercept			94.424	15.281	64.242	124.605	0.000
Neurological status	ECOG Performance Status	0	1/2	-9.574	4.368	-18.204	-0.945	**0.030**
SOSGOQ	Baseline neurological function domain score			-0.801	0.074	-0.948	-0.655	0.000
Tumor information	Visceral/brain metastases	No	Yes	7.112	3.925	-0.645	14.869	**0.072**
**Postoperative AEs as input variable**								
**Variable Group**	**Variable**	**Reference**	**Level**	**Estimate**	**Standard Error**	**Lower Limit**	**Upper Limit**	**P-value**
	Intercept			91.941	15.469	61.387	122.495	0.000
Neurological status	ASIA Impairment Scale	D/E	A/B/C	-10.292	6.104	-22.353	1.770	**0.094**
SOSGOQ	Baseline neurological function domain score			-0.811	0.076	-0.961	-0.661	0.000
Tumor information	Visceral/brain metastases	No	Yes	7.385	3.953	-0.428	15.198	**0.064**

Table XX. Summary of MMRM model for difference of SOSGOQ pain domain score and AE as input variable

						95% Confidence Limit		
**Intraoperative AEs as input variable**								
**Variable Group**	**Variable**	**Reference**	**Level**	**Estimate**	**Standard Error**	**Lower Limit**	**Upper Limit**	**P-value**
	Intercept			68.367	17.019	34.757	101.977	0.000
SOSGOQ	Baseline pain domain score			-0.808	0.070	-0.947	-0.669	0.000
Surgery	Surgical approach	Anterior	Both	-29.515	15.866	-60.869	1.839	0.065
			Posterior	-24.676	11.659	-47.707	-1.645	**0.036**
time	Timepoint	2 Months	6 Months	6.307	2.175	1.988	10.626	0.005
**Postoperative AEs as input variable**								
**Variable Group**	**Variable**	**Reference**	**Level**	**Estimate**	**Standard Error**	**Lower Limit**	**Upper Limit**	**P-value**
	Intercept			69.646	16.656	36.753	102.539	0.000
SOSGOQ	Baseline pain domain score			-0.821	0.070	-0.959	-0.683	0.000
Surgery	Surgical approach	Anterior	Posterior	-23.405	11.508	-46.137	-0.672	**0.044**
time	Timepoint	2 Months	6 Months	6.421	2.181	2.089	10.754	0.004

Table XXI. Summary of MMRM model for difference of SOSGOQ mental domain score and AE as input variable

						95% Confidence Limit		
**Intraoperative AEs as input variable**								
**Variable Group**	**Variable**	**Reference**	**Level**	**Estimate**	**Standard Error**	**Lower Limit**	**Upper Limit**	**P-value**
	Intercept			56.214	19.075	18.553	93.875	0.004
AE	Occurrence of any intraoperative AE	No	Yes	0.048	6.578	-12.957	13.053	0.994
Demographics	Smoking status	No	Yes - previously	-9.738	4.136	-17.908	-1.568	**0.020**
SOSGOQ	Baseline mental domain score			-0.737	0.069	-0.874	-0.600	0.000
**Postoperative AEs as input variable**								
**Variable Group**	**Variable**	**Reference**	**Level**	**Estimate**	**Standard Error**	**Lower Limit**	**Upper Limit**	**P-value**
	Intercept			58.429	18.568	21.769	95.089	0.002
AE	Occurrence of any postoperative AE	No	Yes	-9.650	4.143	-17.834	-1.465	0.021
Demographics	Smoking status	No	Yes - previously	-9.371	4.049	-17.370	-1.373	**0.022**
SOSGOQ	Baseline mental domain score			-0.743	0.067	-0.875	-0.611	0.000

Table XXII. Summary of MMRM model for difference of SOSGOQ social domain score and AE as input variable

						95% Confidence Limit		
**Intraoperative AEs as input variable**								
**Variable Group**	**Variable**	**Reference**	**Level**	**Estimate**	**Standard Error**	**Lower Limit**	**Upper Limit**	**P-value**
	Intercept			64.955	16.336	32.703	97.208	0.000
SOSGOQ	Baseline social domain score			-0.805	0.066	-0.936	-0.675	0.000
Surgery	Number of instrumented levels			-1.773	0.732	-3.220	-0.326	**0.017**
Tumor information	Visceral/brain metastases	No	Yes	8.421	4.656	-0.775	17.617	0.072
time	Timepoint	2 Months	6 Months	4.362	2.256	-0.108	8.832	0.056
**Postoperative AEs as input variable**								
**Variable Group**	**Variable**	**Reference**	**Level**	**Estimate**	**Standard Error**	**Lower Limit**	**Upper Limit**	**P-value**
	Intercept			64.993	15.975	33.452	96.533	0.000
AE	Occurrence of any postoperative AE	No	Yes	-6.986	3.469	-13.841	-0.132	0.046
Neurological status	Total SINS score	Stable: 0-6	Unstable: 13-18	10.143	4.944	0.368	19.918	**0.042**
Surgery	Number of instrumented levels			-1.711	0.721	-3.136	-0.286	**0.019**
time	Timepoint	2 Months	6 Months	4.564	2.255	0.097	9.032	0.045
